# Supplementary material for: LC-MS Metabolite Profiling and the Hypoglycemic Activity of Morus alba L. Extracts
Source: Molecules. 2022 Aug 23;27(17):5360. doi: 10.3390/molecules27175360 (PMC9457631; doi:10.3390/molecules27175360)
Supplement: Supplementary file 1 [file molecules-27-05360-s001.zip › molecules-1830219-supplementary.pdf]

molecules

Supplementary Materials

LC-MS Metabolite Profiling and the Hypoglycemic Activity of *Morus alba* L. Extracts

Qing Yi-Jun Zhou [1,2], Xin Liao [2], Hao-Ming Kuang [2], Jia-Yu Li [2] and Shui-Han Zhang [1,2,*]

[1] Science and Technology Innovation Center, Hunan University of Chinese Medicine, 410208 Changsha, China
[2] Institute of Chinese Materia Medica, Hunan Academy of Chinese Medicine, 410013 Changsha, China
* Correspondence: zhangshuihan0220@126.com; Tel.: +86-731-84686656

Figure. S1 the MS/MS spectra of compounds 1–60.

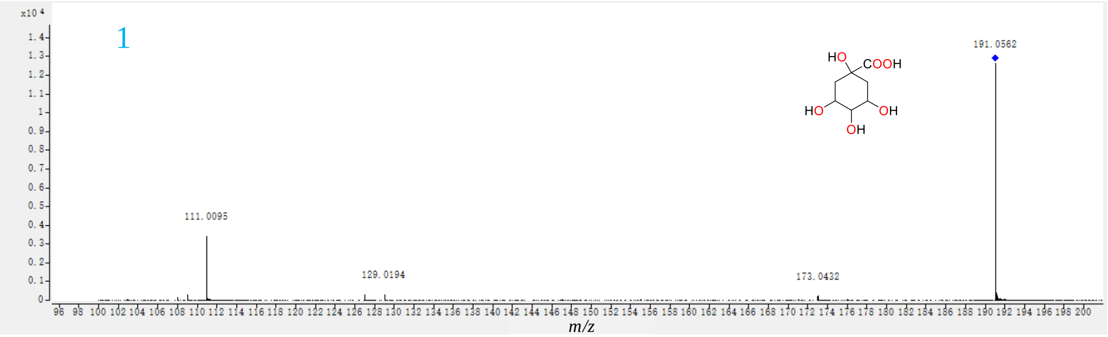

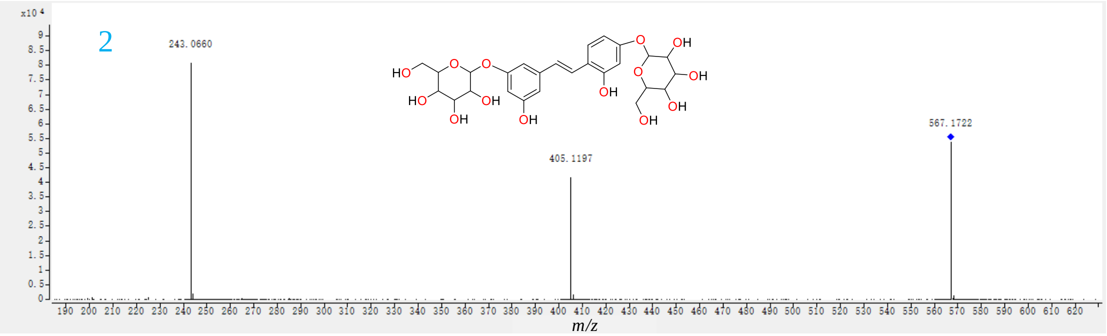

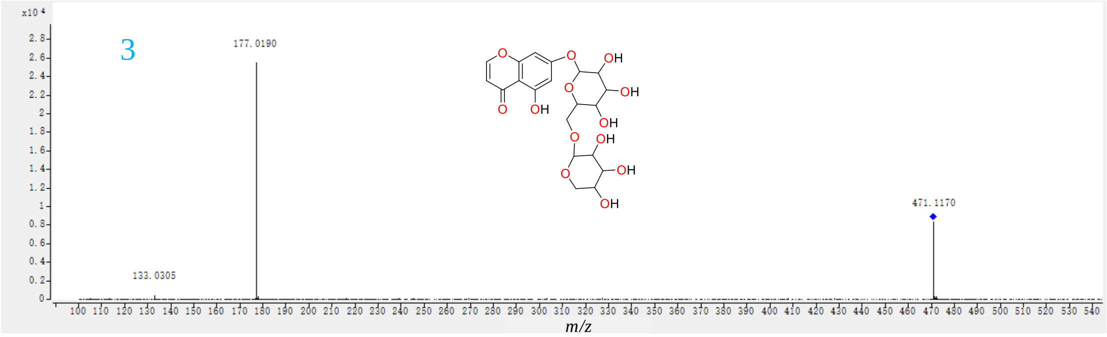

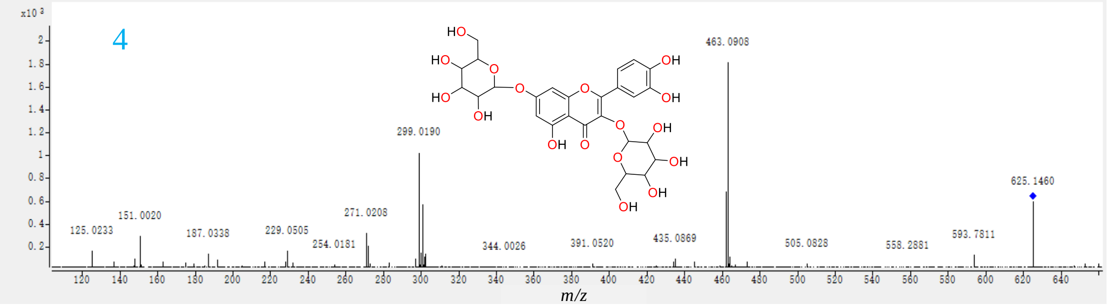

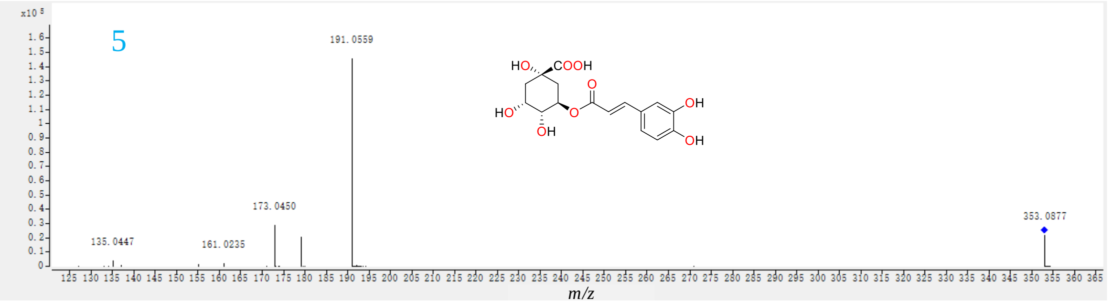
x10 5
5
191.0559
173.0450
135.0447
161.0235
353.0877
HO
COOH
O
OH
OH
m/z

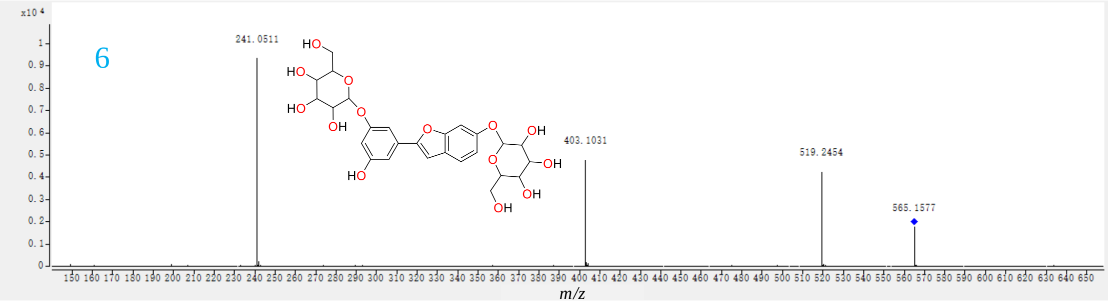
x10 4
6
241.0511
403.1031
519.2454
565.1577
HO
OH
m/z

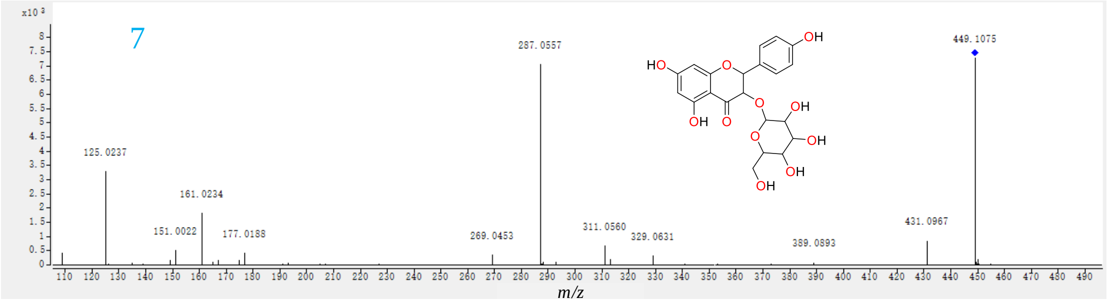
x10 3
7
287.0557
449.1075
125.0237
161.0234
151.0022
177.0188
269.0453
311.0560
329.0631
389.0893
431.0967
HO
OH
m/z

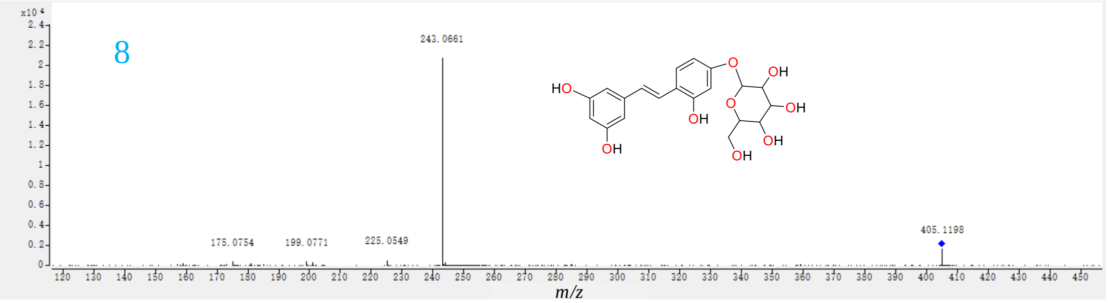
x10 4
8
243.0661
175.0754
199.0771
225.0549
405.1198
HO
OH
m/z

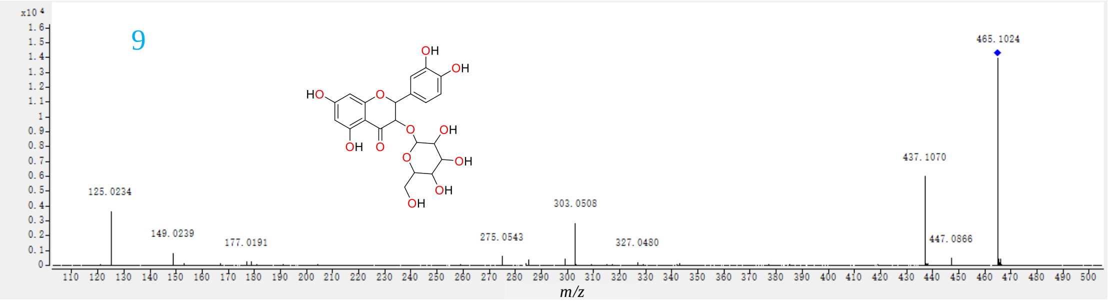
x10 4
9
465.1024
437.1070
125.0234
149.0239
177.0191
275.0543
303.0508
327.0480
447.0866
HO
OH
m/z

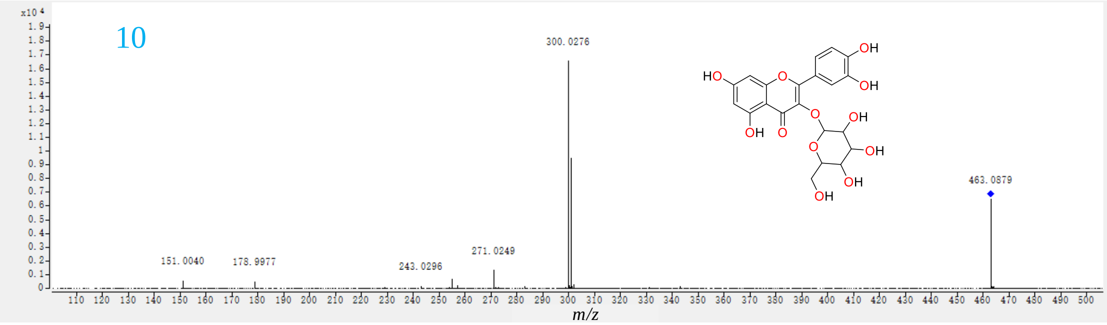
10
300.0276
463.0879
151.0040
178.9977
243.0296
271.0249
m/z

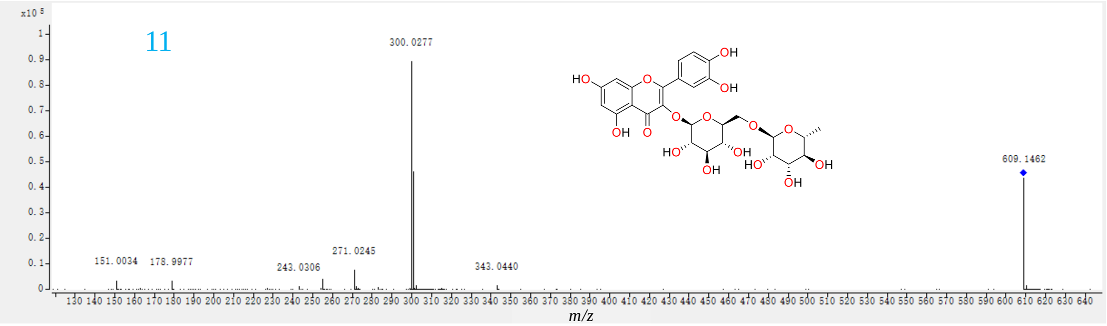
11
300.0277
609.1462
151.0034
178.9977
243.0306
271.0245
343.0440
m/z

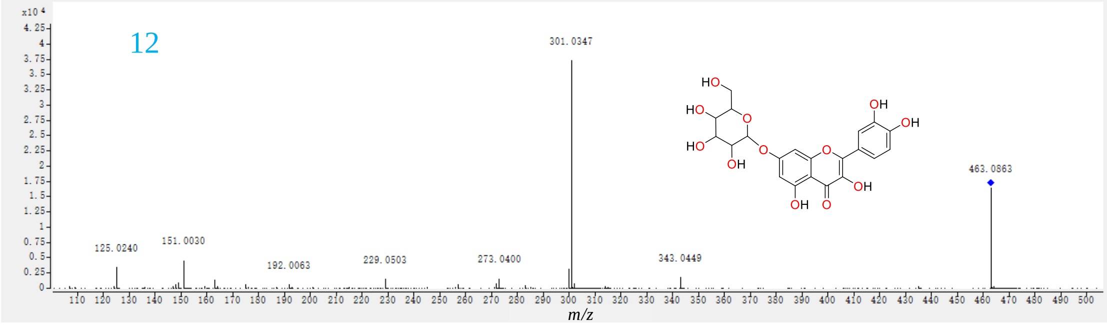
12
301.0347
463.0863
125.0240
151.0030
192.0063
229.0503
273.0400
343.0449
m/z

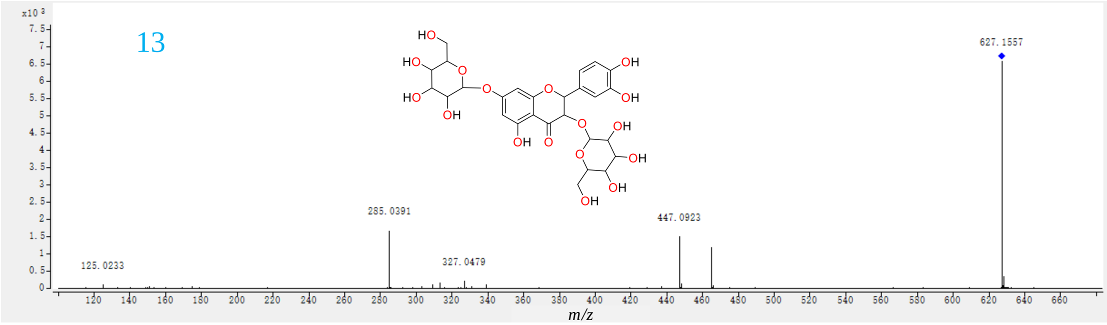
13
627.1557
125.0233
285.0391
327.0479
447.0923
m/z

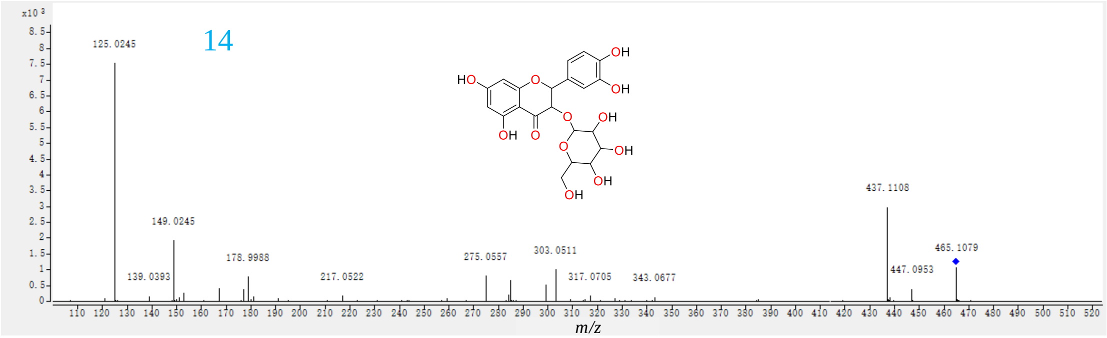
14
125.0245
149.0245
139.0393
178.9988
217.0522
275.0557
303.0511
317.0705
343.0677
437.1108
447.0953
465.1079
m/z

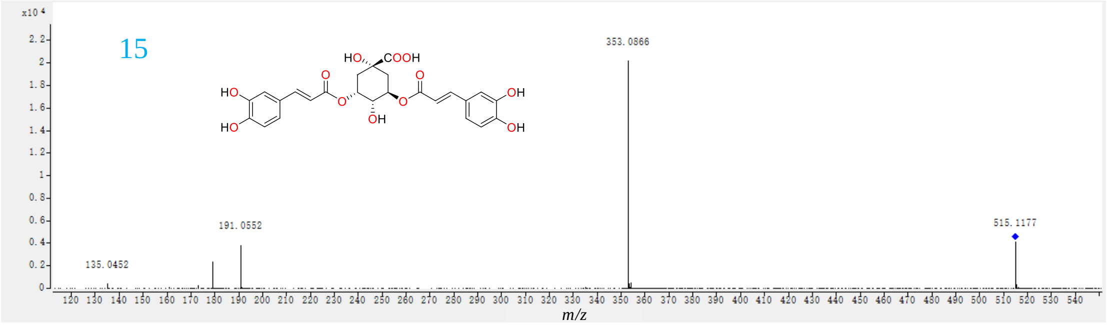
15
x10 4
353.0866
191.0552
135.0452
515.1177
m/z

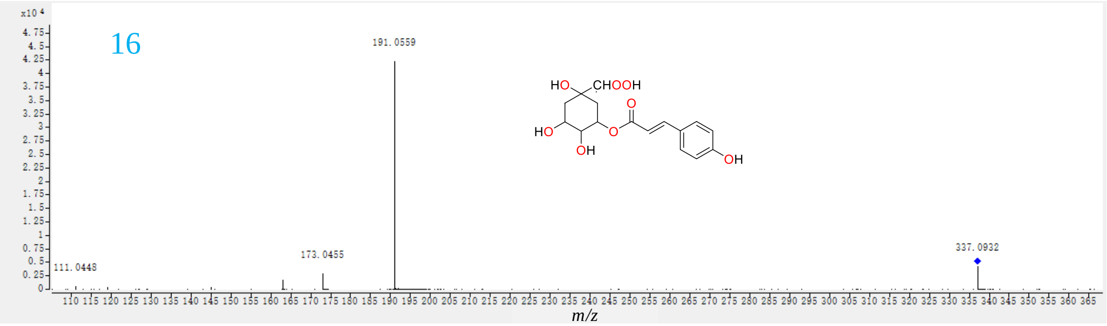
16
x10 4
191.0559
173.0455
111.0448
337.0932
m/z

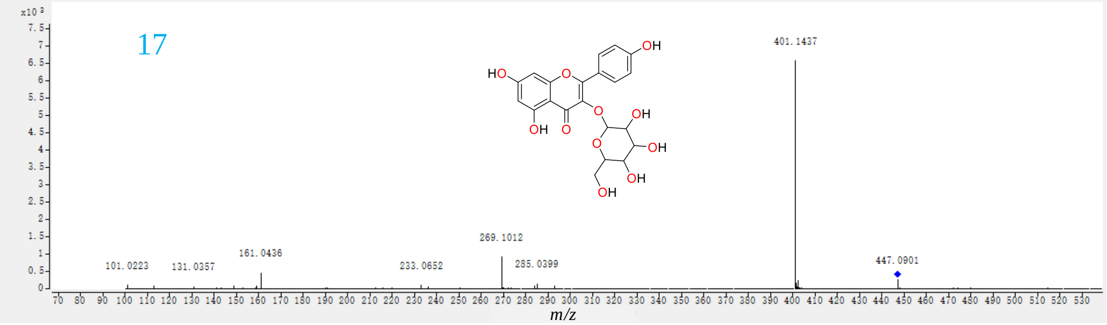
17
x10 3
401.1437
269.1012
285.0399
233.0652
161.0436
131.0357
101.0223
447.0901
m/z

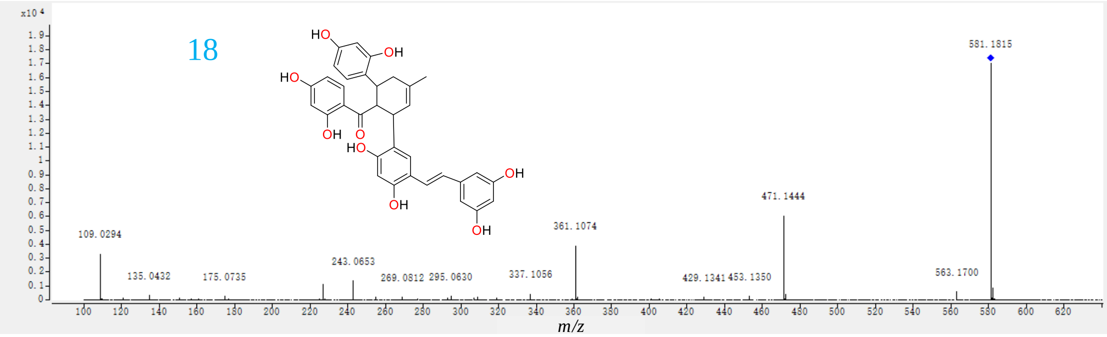
18
x10 4
581.1815
471.1444
361.1074
109.0294
135.0432
175.0735
243.0653
269.0812 295.0630
337.1056
429.1341 453.1350
563.1700
m/z

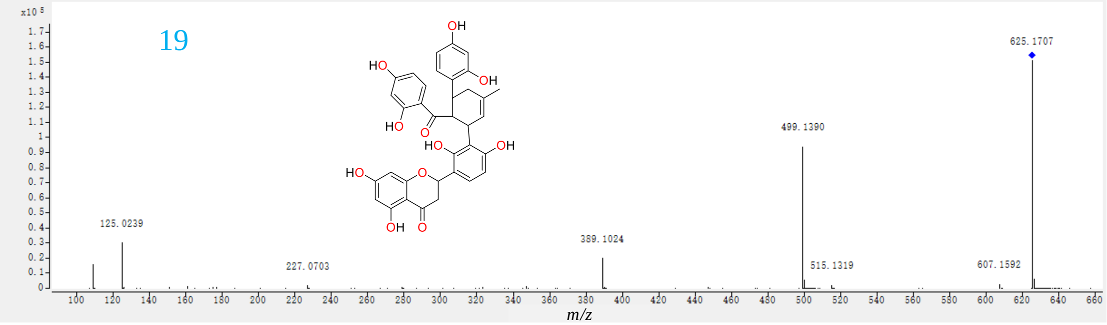
19
x10 5
625.1707
499.1390
125.0239
227.0703
389.1024
515.1319
607.1592
m/z

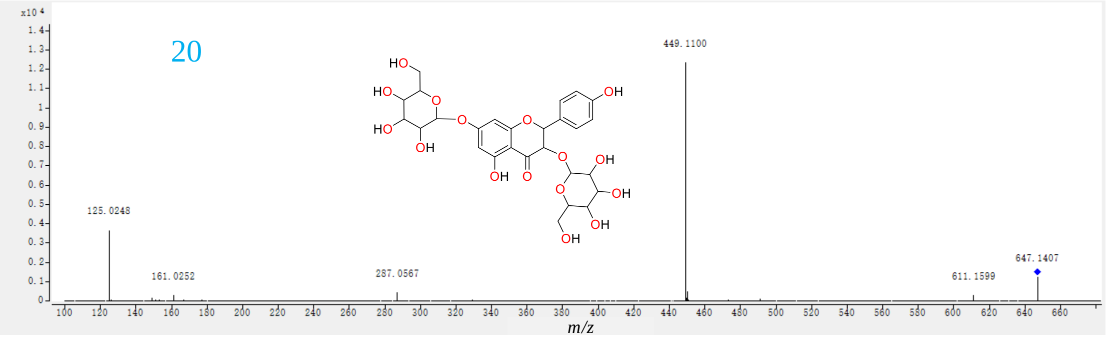
20
449.1100
125.0248
161.0252
287.0567
611.1599
647.1407
x10 4
m/z

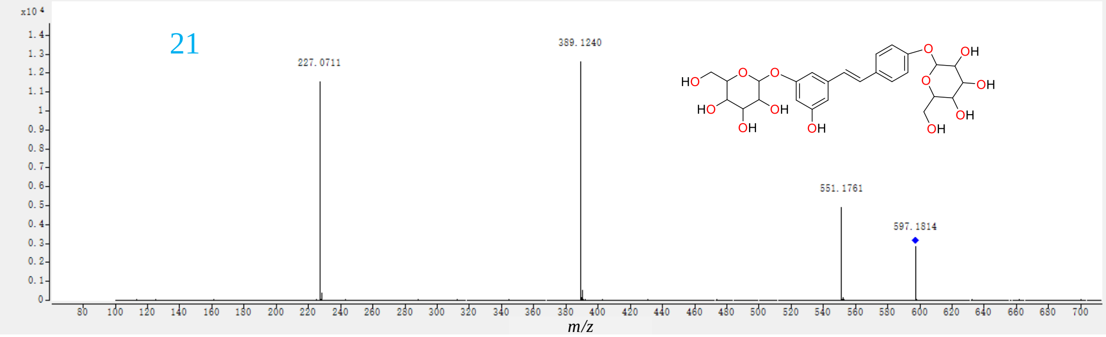
21
227.0711
389.1240
551.1761
597.1814
x10 4
m/z

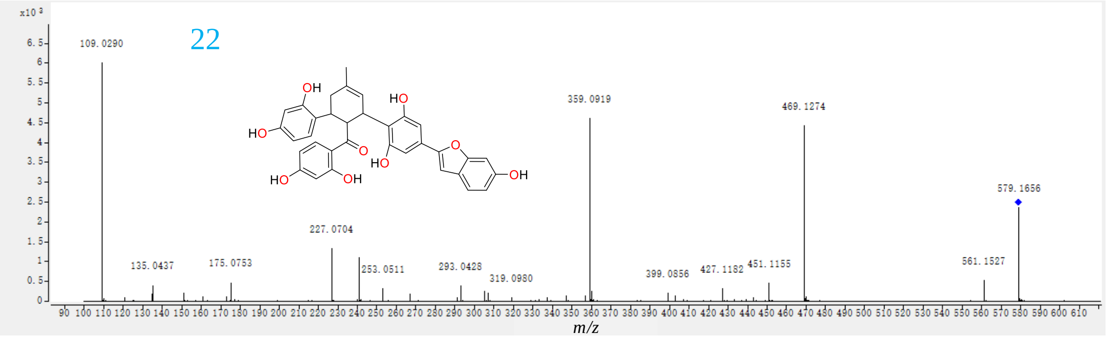
22
109.0290
359.0919
469.1274
579.1656
227.0704
135.0437
175.0753
253.0511
293.0428
319.0980
399.0856
427.1182
451.1155
561.1527
x10 3
m/z

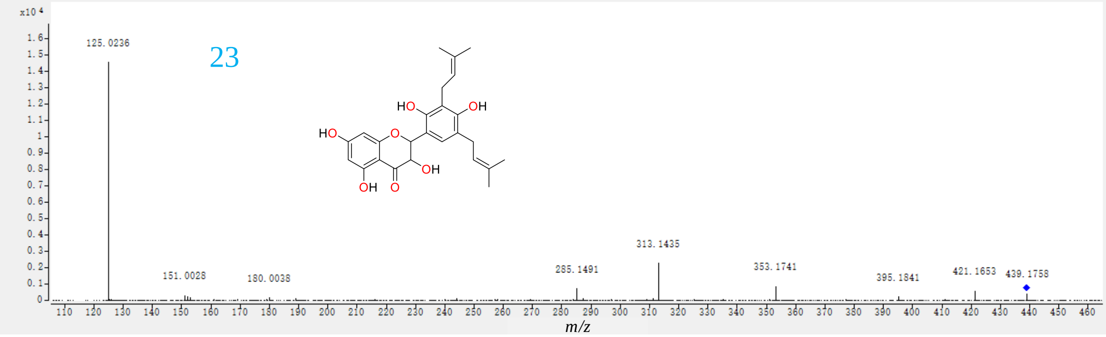
23
125.0236
151.0028
180.0038
285.1491
313.1435
353.1741
395.1841
421.1653
439.1758
x10 4
m/z

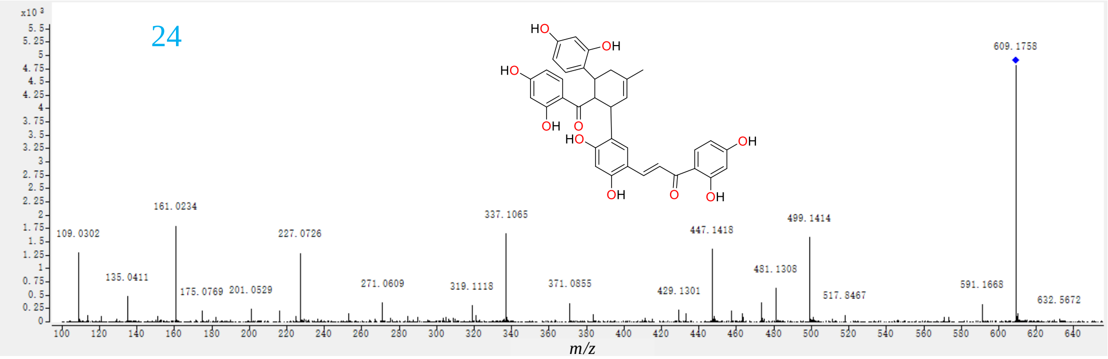
24
x10 3
609.1758
161.0234
109.0302
135.0411
175.0769
201.0529
227.0726
271.0609
319.1118
337.1065
371.0855
429.1301
447.1418
481.1308
499.1414
517.8467
591.1668
632.5672
m/z

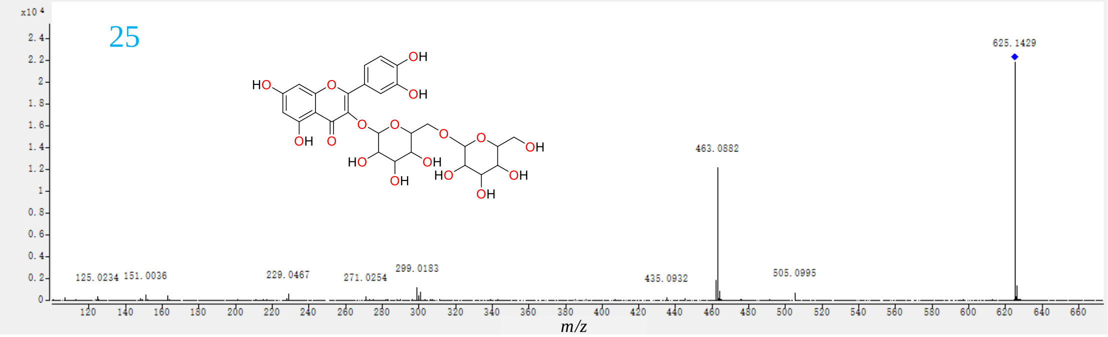
25
x10 4
625.1429
463.0882
125.0234
151.0036
229.0467
271.0254
299.0183
435.0932
505.0995
m/z

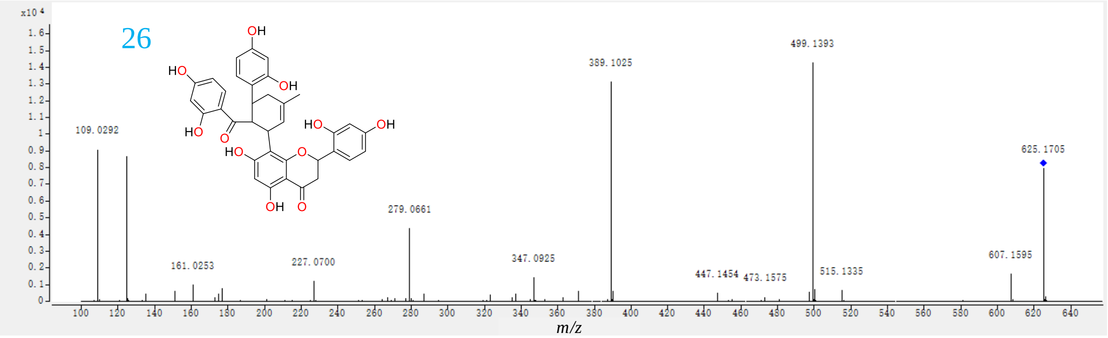
26
x10 4
109.0292
161.0253
227.0700
279.0661
347.0925
389.1025
447.1454
473.1575
499.1393
515.1335
607.1595
625.1705
m/z

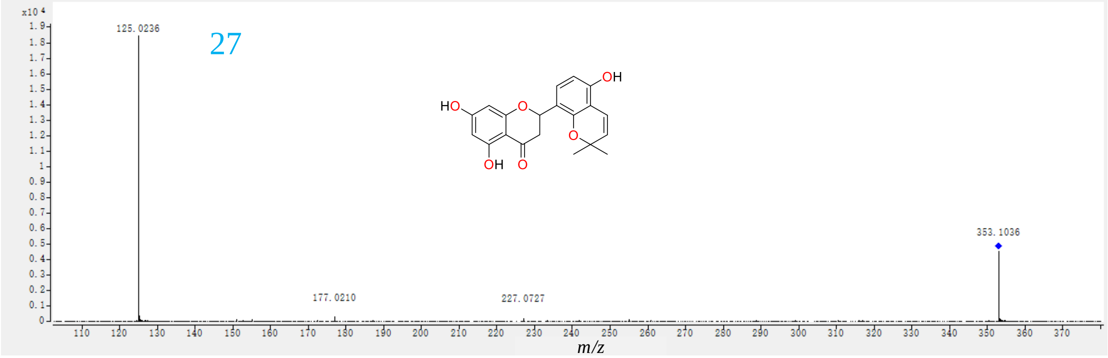
27
x10 4
125.0236
177.0210
227.0727
353.1036
m/z

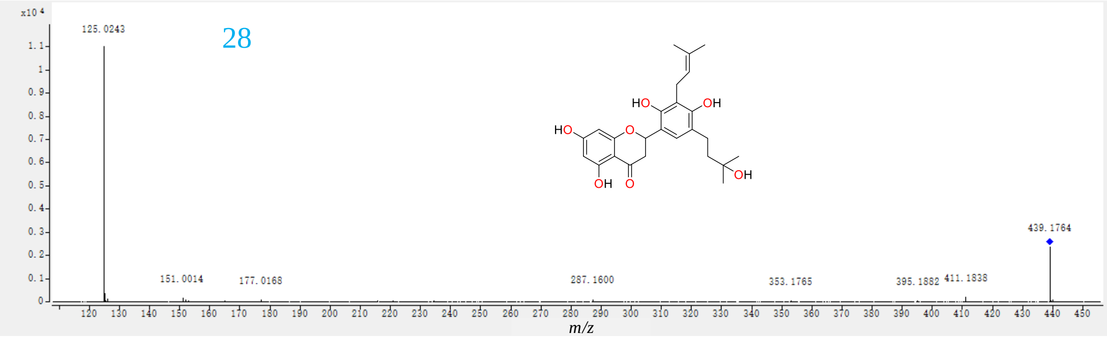
28
x10 4
125.0243
151.0014
177.0168
287.1600
353.1765
395.1882
411.1838
439.1764
m/z

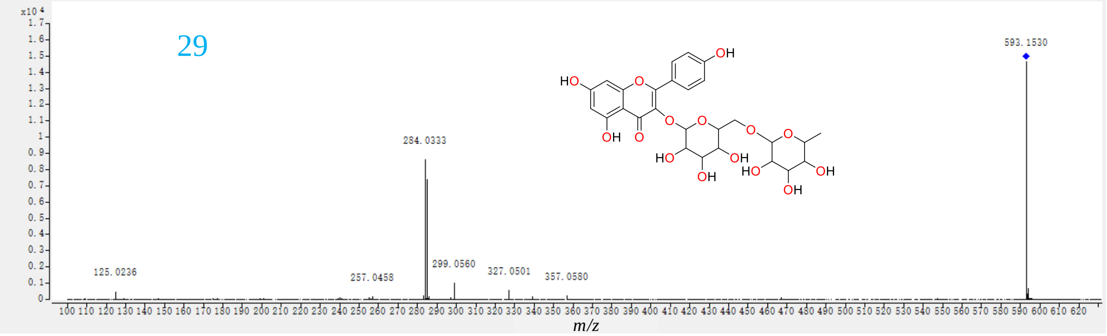
29
x10 4
125.0236
257.0458
284.0333
299.0560
327.0501
357.0580
593.1530
m/z

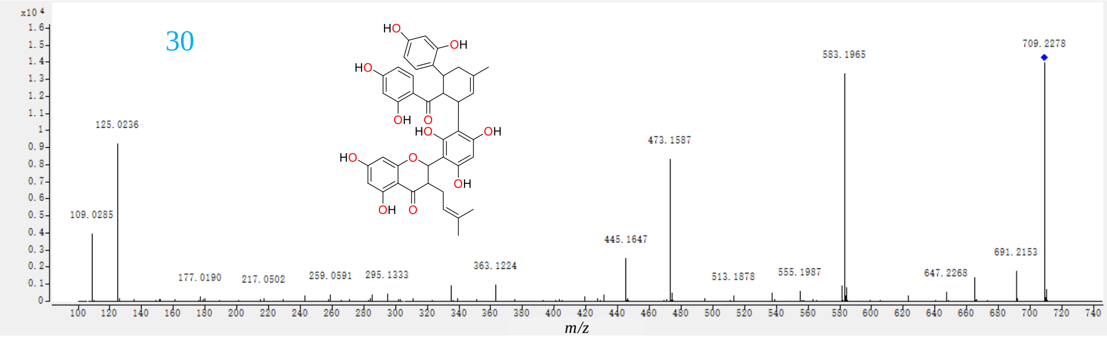
30
x10 4
109.0285
125.0236
177.0190
217.0502
259.0591
295.1333
363.1224
445.1647
473.1587
513.1878
555.1987
583.1965
647.2268
691.2153
709.2278
m/z

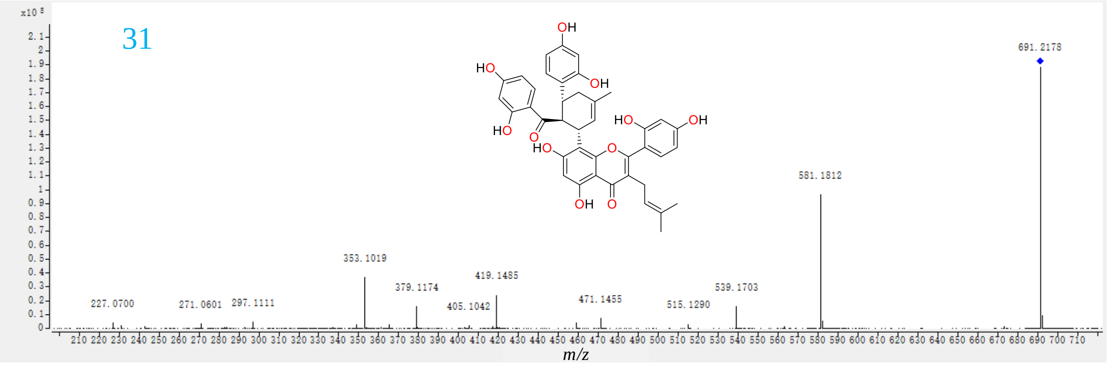
31
x10 5
227.0700
271.0601
297.1111
353.1019
379.1174
405.1042
419.1485
471.1455
515.1290
539.1703
581.1812
691.2178
m/z

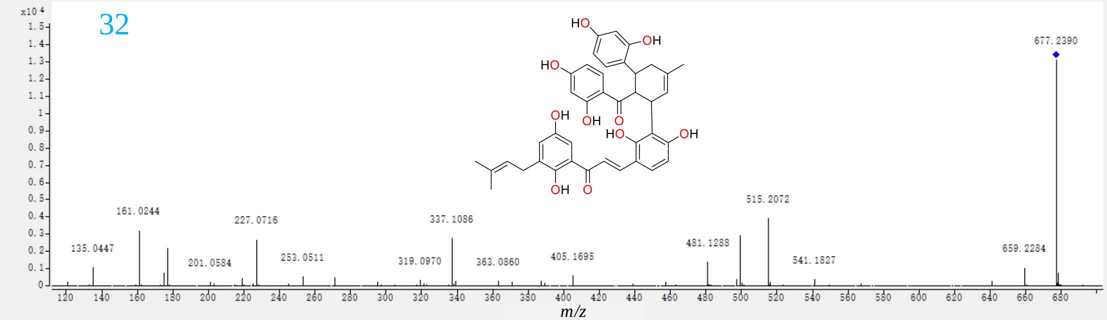
32
677.2390
515.2072
161.0244
227.0716
337.1086
135.0447
201.0584
253.0511
319.0970
363.0860
405.1695
481.1288
541.1827
659.2284
m/z

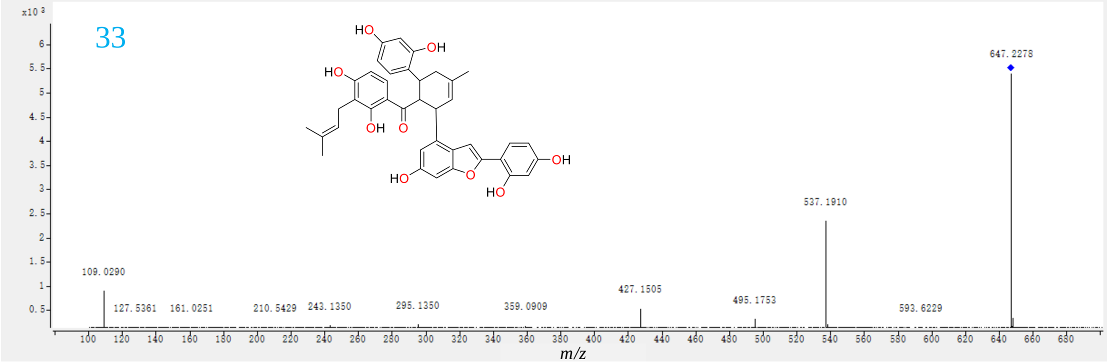
33
647.2278
537.1910
109.0290
427.1505
495.1753
127.5361
161.0251
210.5429
243.1350
295.1350
359.0909
593.6229
m/z

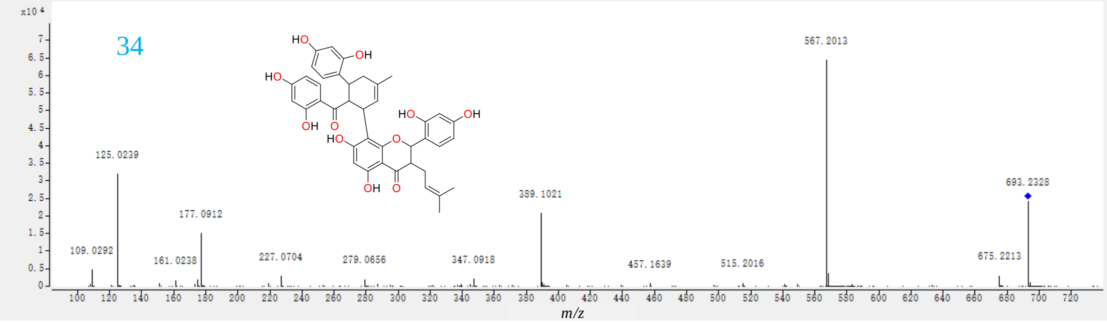
34
567.2013
693.2328
125.0239
177.0912
389.1021
109.0292
161.0238
227.0704
279.0656
347.0918
457.1639
515.2016
675.2213
m/z

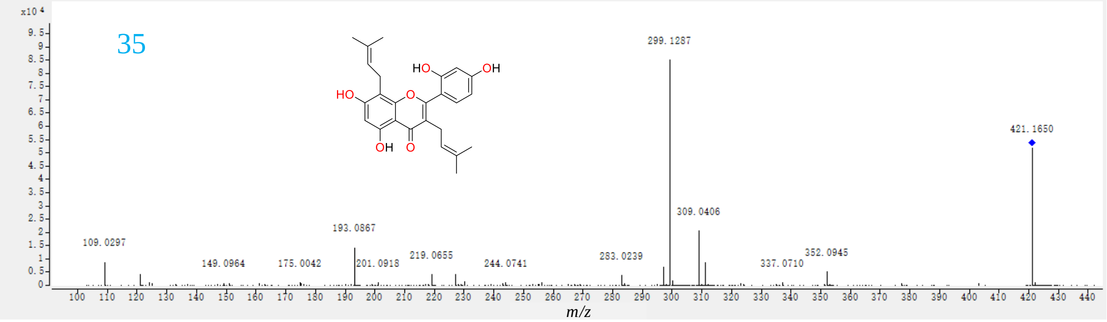
35
299.1287
421.1650
309.0406
193.0867
109.0297
149.0964
175.0042
201.0918
219.0655
244.0741
283.0239
337.0710
352.0945
m/z

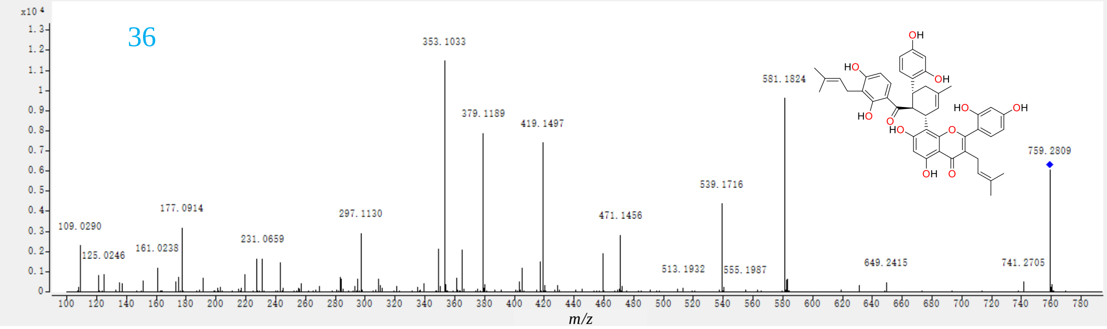
36
353.1033
581.1824
379.1189
419.1497
759.2809
539.1716
177.0914
297.1130
471.1456
109.0290
231.0659
161.0238
125.0246
513.1932
555.1987
649.2415
741.2705
m/z

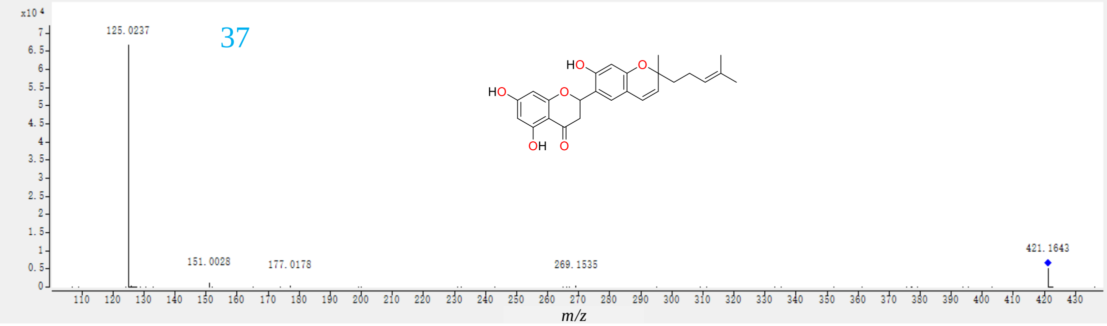
37
125.0237
421.1643
151.0028
177.0178
269.1535
m/z

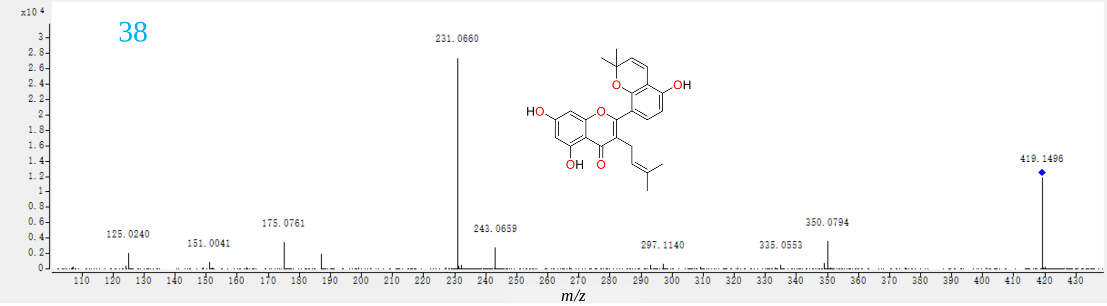
38
231.0660
419.1496
175.0761
243.0659
350.0794
125.0240
151.0041
297.1140
335.0553
m/z

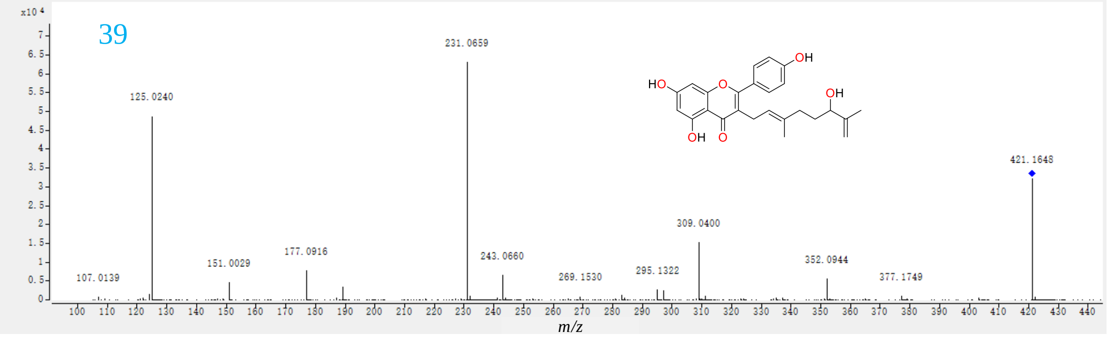
39
231.0659
125.0240
421.1648
309.0400
177.0916
243.0660
151.0029
352.0944
295.1322
107.0139
269.1530
377.1749
m/z

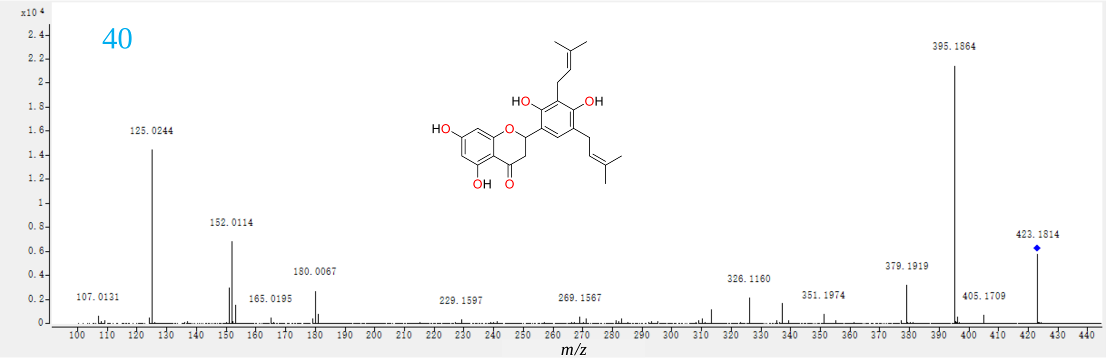
x10 4
40
395.1864
125.0244
152.0114
423.1814
379.1919
180.0067
326.1160
107.0131
165.0195
229.1597
269.1567
351.1974
405.1709
m/z

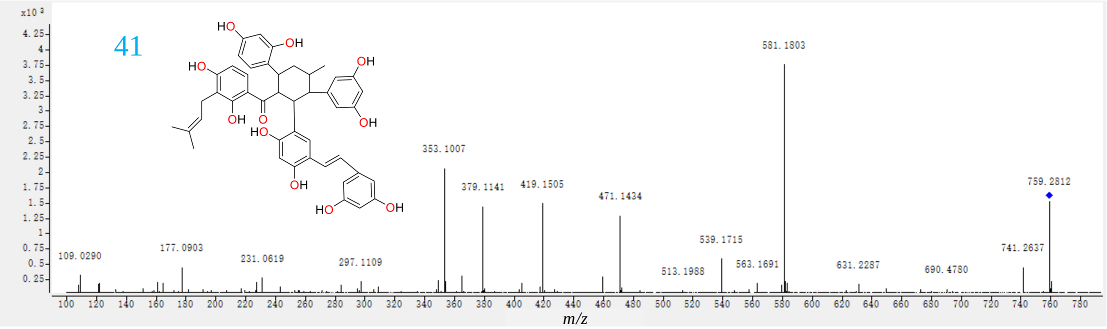
x10 3
41
581.1803
353.1007
379.1141
419.1505
471.1434
759.2812
539.1715
109.0290
177.0903
231.0619
297.1109
513.1988
563.1691
631.2287
690.4780
741.2637
m/z

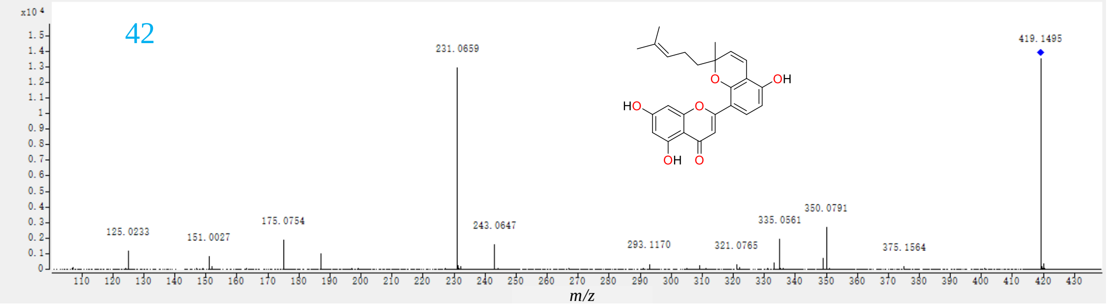
x10 4
42
231.0659
419.1495
125.0233
151.0027
175.0754
243.0647
293.1170
321.0765
335.0561
350.0791
375.1564
m/z

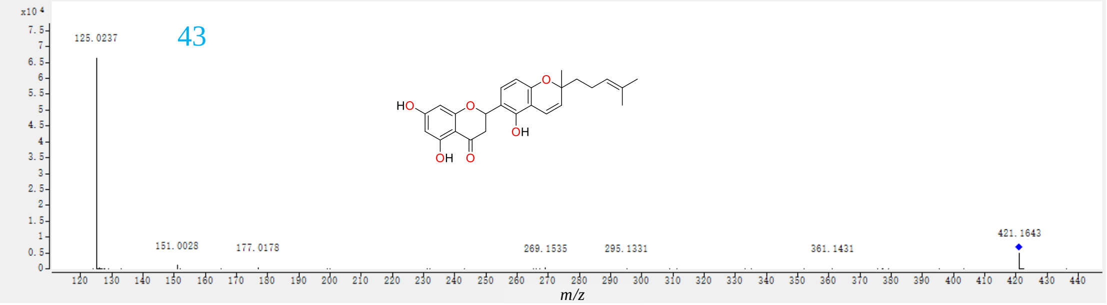
x10 4
125.0237
43
151.0028
177.0178
269.1535
295.1331
361.1431
421.1643
m/z

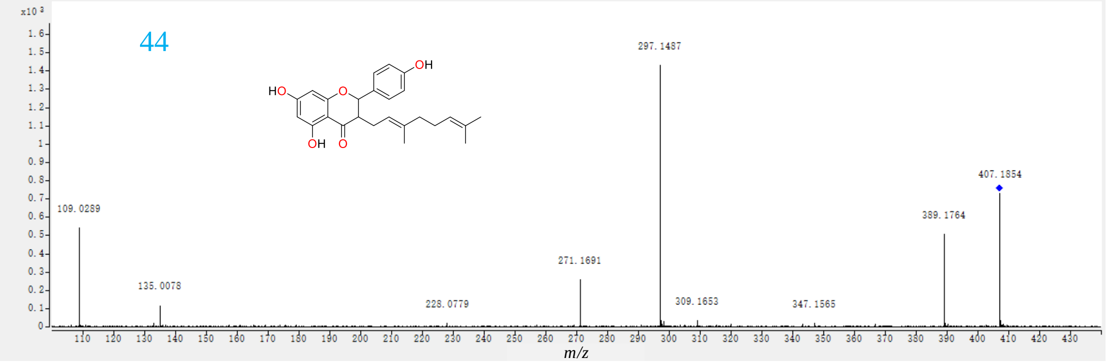
44
297.1487
407.1854
389.1764
109.0289
271.1691
135.0078
228.0779
309.1653
347.1565
m/z

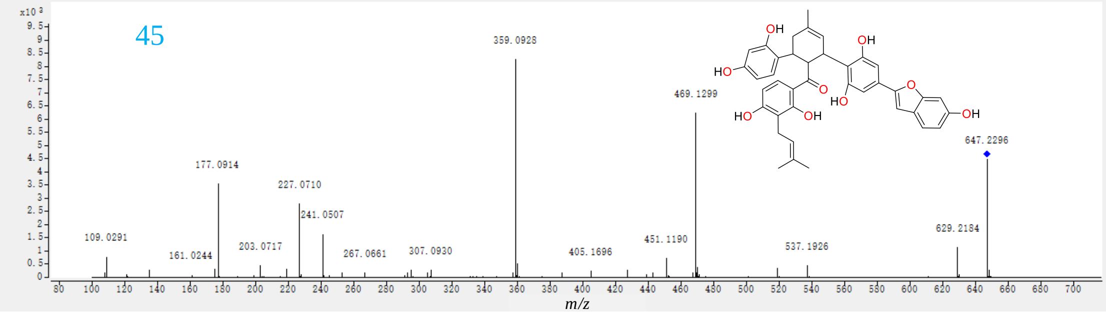
45
359.0928
469.1299
647.2296
177.0914
227.0710
241.0507
629.2184
109.0291
161.0244
203.0717
267.0661
307.0930
405.1696
451.1190
537.1926
m/z

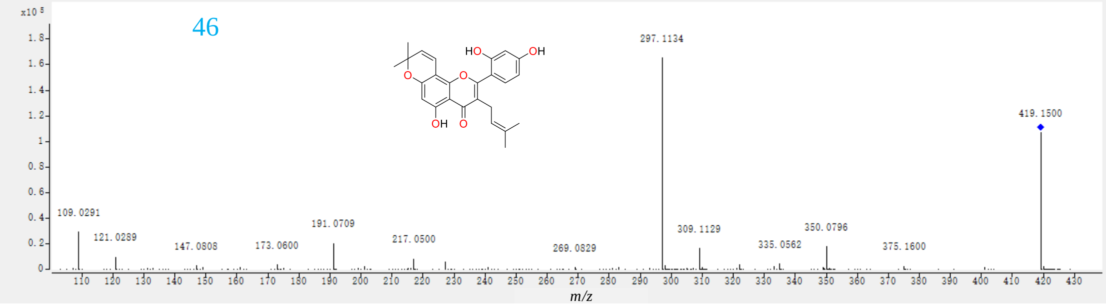
46
297.1134
419.1500
109.0291
121.0289
147.0808
173.0600
191.0709
217.0500
269.0829
309.1129
335.0562
350.0796
375.1600
m/z

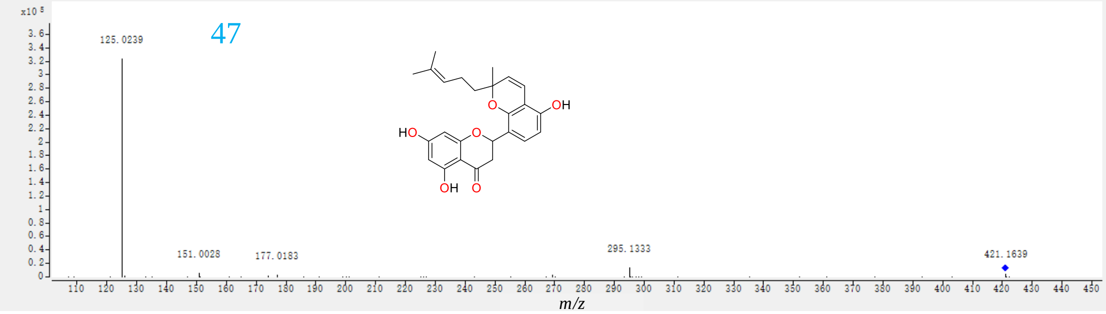
47
125.0239
151.0028
177.0183
295.1333
421.1639
m/z

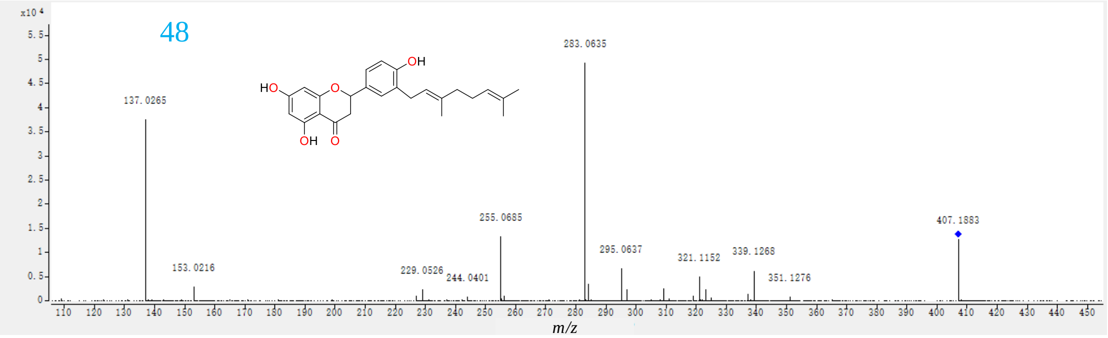
48
137. 0265
153. 0216
229. 0526
244. 0401
255. 0685
283. 0635
295. 0637
321. 1152
339. 1268
351. 1276
407. 1883
m/z

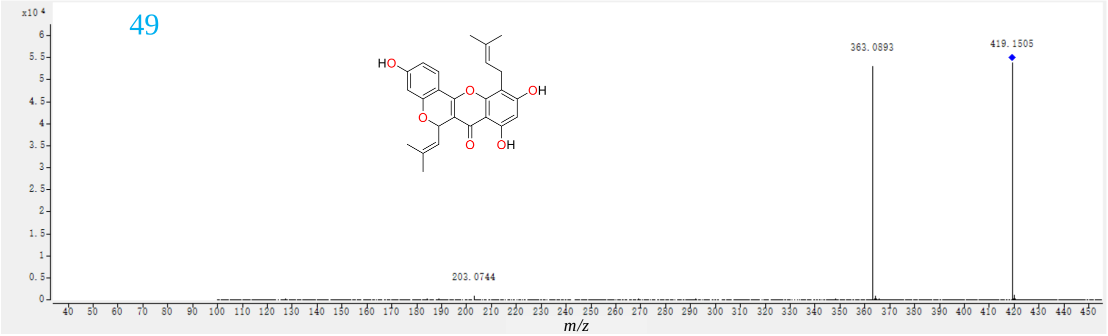
49
203. 0744
363. 0893
419. 1505
m/z

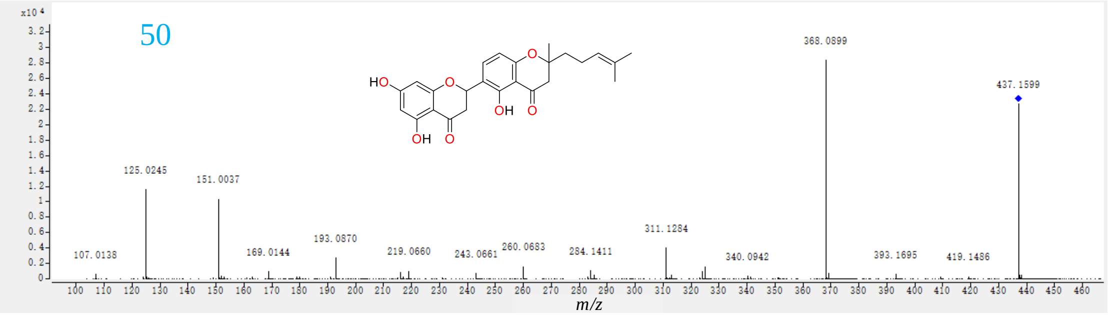
50
107. 0138
125. 0245
151. 0037
169. 0144
193. 0870
219. 0660
243. 0661
260. 0683
284. 1411
311. 1284
340. 0942
368. 0899
393. 1695
419. 1486
437. 1599
m/z

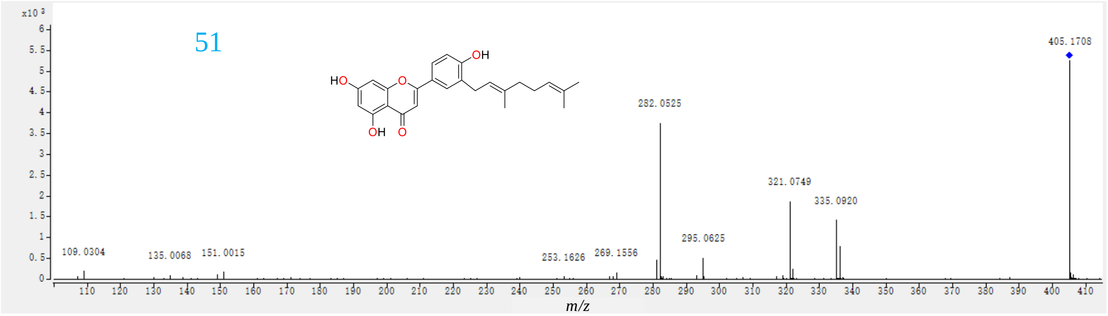
51
109. 0304
135. 0068
151. 0015
253. 1626
269. 1556
282. 0525
295. 0625
321. 0749
335. 0920
405. 1708
m/z

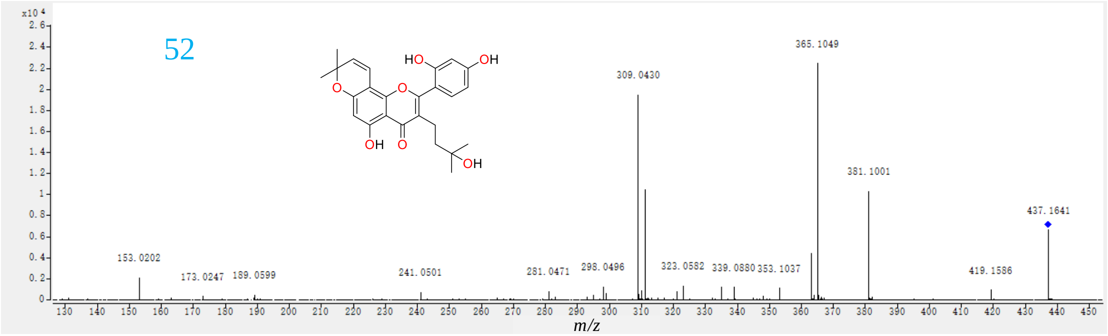
52
153. 0202
173. 0247
189. 0599
241. 0501
281. 0471
298. 0496
309. 0430
323. 0582
339. 0880
353. 1037
365. 1049
381. 1001
419. 1586
437. 1641
m/z

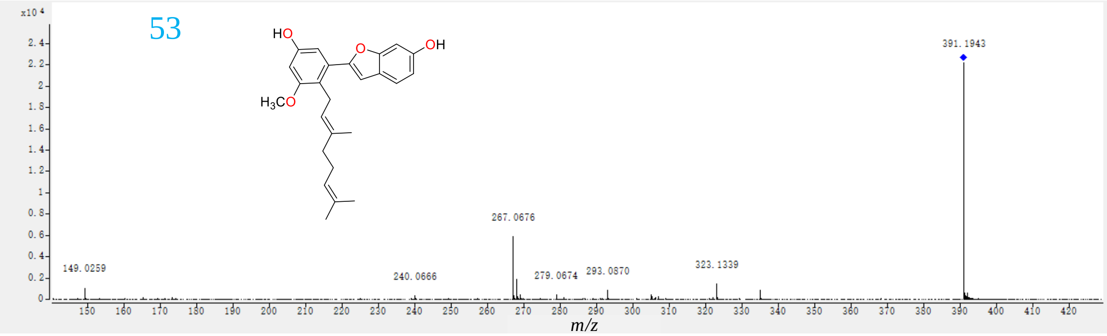
53
x10 4
391.1943
267.0676
149.0259
240.0666
279.0674
293.0870
323.1339
HO
OH
H_3CO
m/z

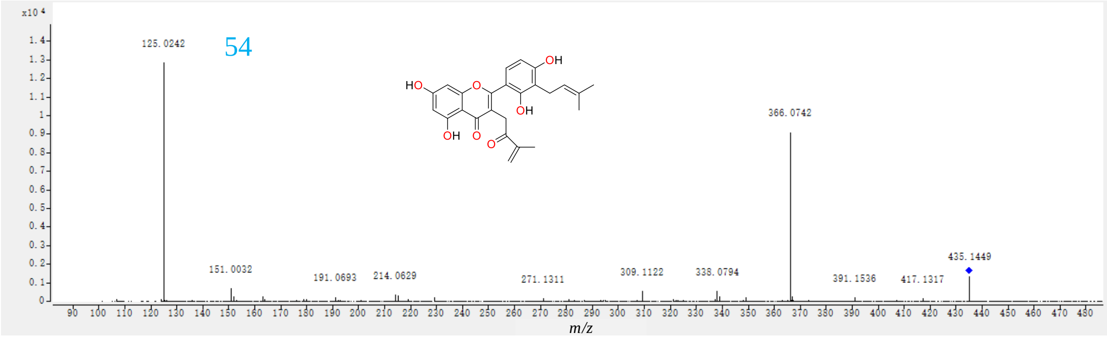
54
x10 4
125.0242
366.0742
151.0032
191.0693
214.0629
271.1311
309.1122
338.0794
391.1536
417.1317
435.1449
HO
OH
m/z

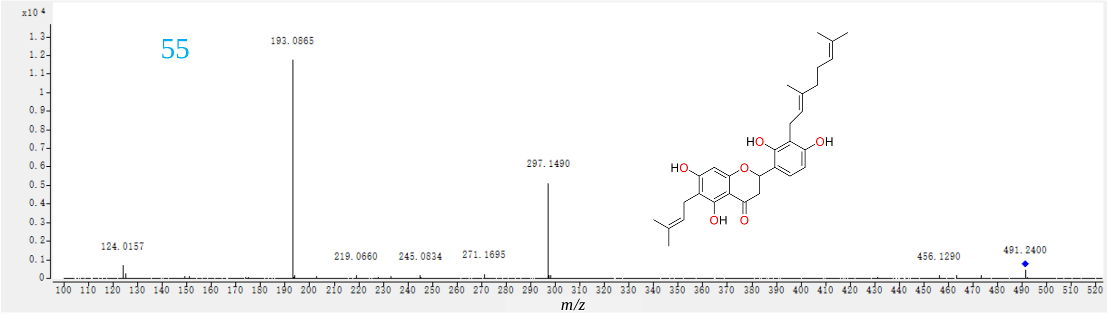
55
x10 4
193.0865
297.1490
124.0157
219.0660
245.0834
271.1695
456.1290
491.2400
HO
OH
m/z

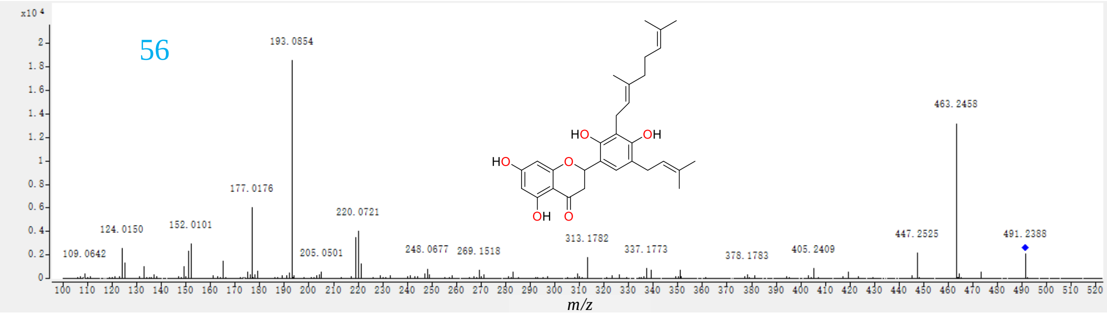
56
x10 4
193.0854
463.2458
177.0176
220.0721
124.0150
152.0101
109.0642
205.0501
248.0677
269.1518
313.1782
337.1773
378.1783
405.2409
447.2525
491.2388
HO
OH
m/z

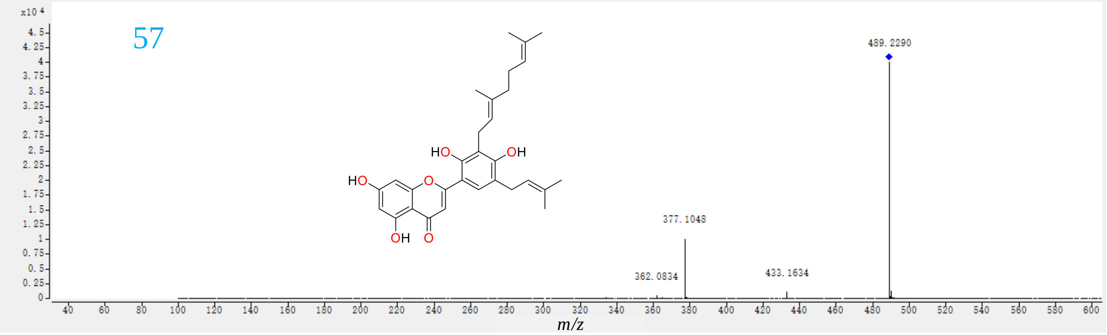
57
x10 4
489.2290
377.1048
362.0834
433.1634
HO
OH
m/z

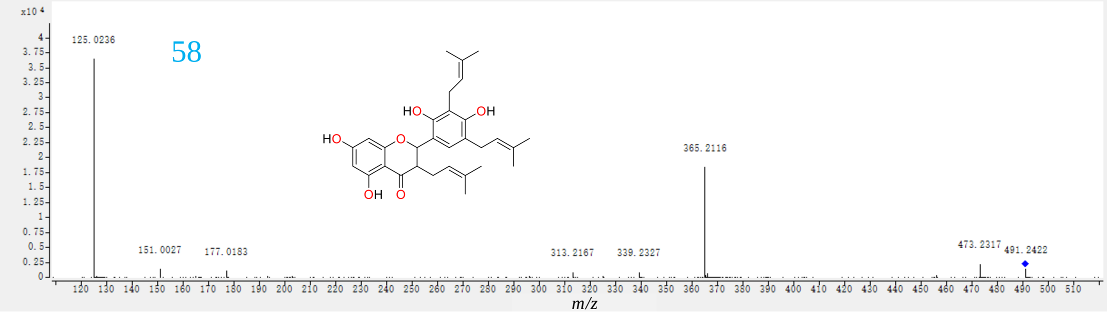
58
x10 4
125.0236
151.0027
177.0183
313.2167
339.2327
365.2116
473.2317
491.2422
HO
OH
m/z

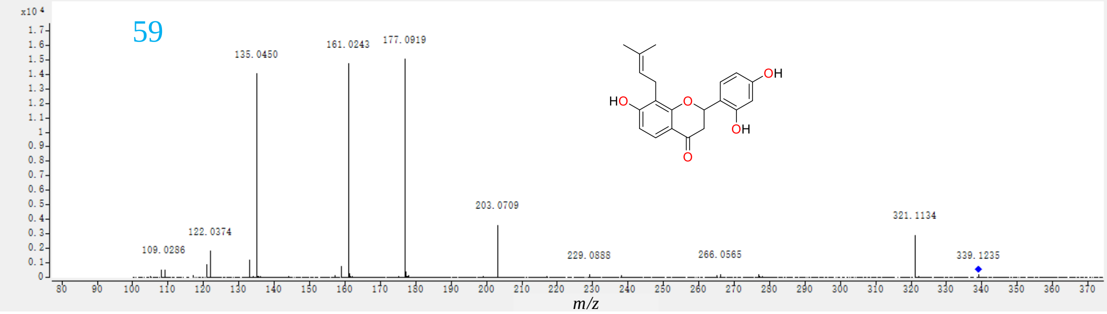
59
x10 4
109.0286
122.0374
135.0450
161.0243
177.0919
203.0709
229.0888
266.0565
321.1134
339.1235
HO
OH
m/z

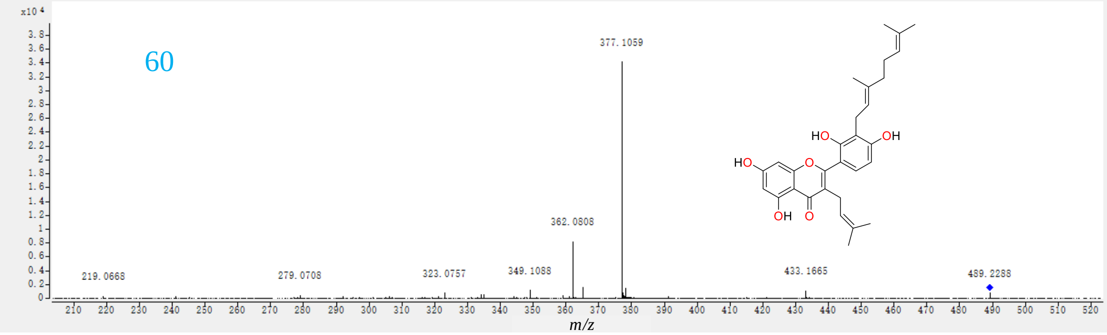
60
x10 4
219.0668
279.0708
323.0757
349.1088
362.0808
377.1059
433.1665
489.2288
HO
OH
m/z

Table S1 The database of reported compounds from the genus of *Morus*.

No.	Name	Molecular formula	[M+H]+	[M-H]-	Structure
1	Lutin	$C_{27}H_{30}O_{16}$	611.1612	609.1456	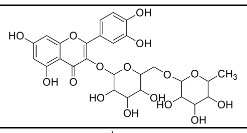
2	Mulberrofuran F	$C_{39}H_{34}O_{9}$	647.6898	645.6739	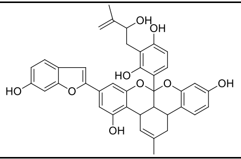
3	Isoquercitrin	$C_{21}H_{20}O_{12}$	465.1033	463.0877	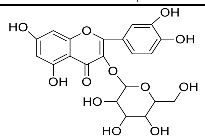
4	Hyperosidine	$C_{21}H_{20}O_{11}$	449.1078	447.0927	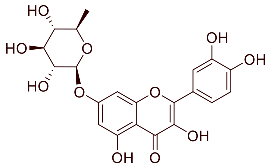
5	Astragalin	$C_{21}H_{20}O_{11}$	449.1084	447.0927	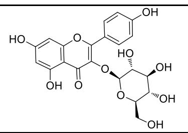
6	Morin	$C_{15}H_{12}O_{8}$	321.0610	319.0454	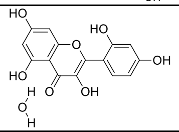
7	Keampferol	$C_{15}H_{10}O_{6}$	287.0556	285.0399	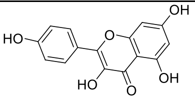
8	Muberroside A1	$C_{26}H_{32}O_{14}$	569.1870	567.1714	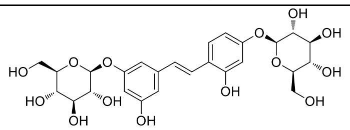
9	Muberroside E	C_{26} $H_{32}O_{13}$	553.1921	551.1765	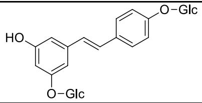
10	Muberroside F	$C_{26}H_{30}O_{14}$	567.1724	565.1557	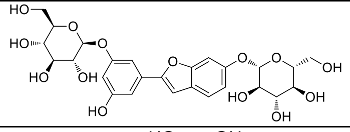
11	Kelampayoside A	$C_{20}H_{30}O_{13}$	479.1765	477.1608	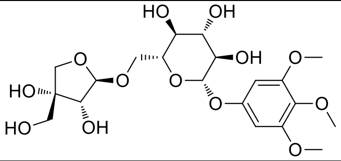
12	Oxyresveratrol	$C_{14}H_{12}O4$	245.0814	243.0657	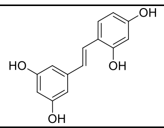
13	DNJ	$C_{6}H_{13}NO_{4}$	164.0923	162.0766	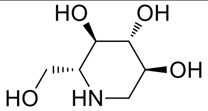

14	Resveratrol	$C_{14}H_{12}O_3$	229.0865	227.0708	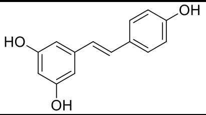
15	Cyanidin-3-*O*-glucoside	$C_{21}H_{21}O_{11}$	449.1078	447.022	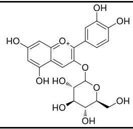
16	Keracyanin	$C_{27}H_{31}O_{15}$	595.1657	593.1501	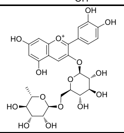
17	1-[5-(2-formlfuryl)methyl]dihydrogen2-hydroxypropane-1,2,3-tricarboxylate2,3-diethyl ester	$C_{16}H_{20}O_9$	357.1186	355.1029	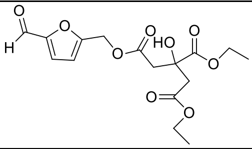
18	3,4-(dimethoxyhydro)cinnamic acid	$C_{10}H_{10}O_4$	195.0657	193.0501	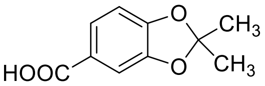
19	Salicylic acid	$C_7H_6O_3$	139.0395	137.0239	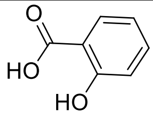
20	Succinic acid	$C_4H_6O_4$	119.0344	117.0188	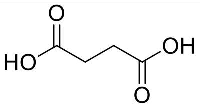
21	Daucoside	$C_{35}H_{60}O_6$	577.4468	575.4312	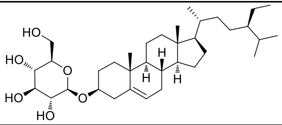
22	Dulcitol	$C_6H_{14}O_6$	183.0869	181.0712	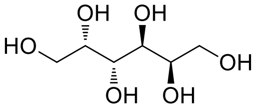
23	Aurantiamide	$C_{25}H_{26}N_2O_3$	403.2022	401.1865	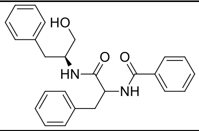
24	Cytochalasin B	$C_{29}H_{37}NO_5$	480.2750	478.2593	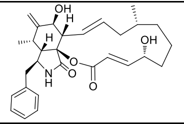
25	β-sitosterol	$C_{29}H_{50}O$	415.3940	413.3783	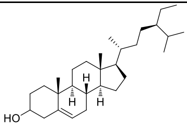
26	7β-hydroxy-sitosterol	$C_{29}H_{50}O_2$	431.3880	429.3733	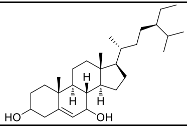
27	(22E)-24-methyl-5α-cholesta-7,22-diene-3β,5α,6β-triol	$C_{28}H_{46}O_3$	431.3525	429.3369	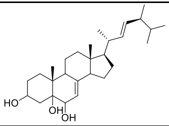
28	(22E)-5α,8α-epidioxyergosta-6,22-dien-3β-ol	$C_{28}H_{44}O_3$	429.3369	427.3212	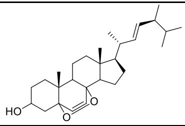

29	Chalcomoracin	$C_{39}H_{36}O_9$	649.2438	647.2281	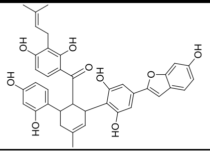
30	Morachalcone A	$C_{20}H_{20}O_5$	341.1389	339.1232	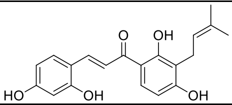
31	Isobavachalcone	$C_{20}H_{20}O_4$	325.1440	323.1283	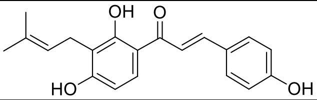
32	Norartocarpetin	$C_{15}H_{10}O_6$	287.0556	285.0399	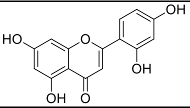
33	Mulberrofuran F1	$C_{39}H_{34}O_9$	647.2281	645.2125	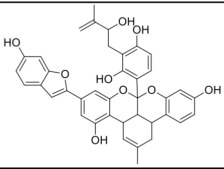
34	Isoquercitrin	$C_{21}H_{20}O_{12}$	465.1033	463.0877	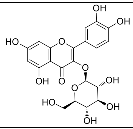
35	Mulberrin	$C_{25}H_{26}O_6$	423.1808	421.1651	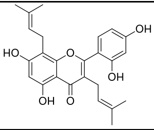
36	Dihydromorin	$C_{15}H_{12}O_7$	305.0661	303.0505	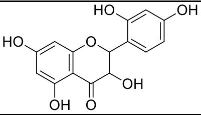
37	Cyclomulberrin	$C_{25}H_{24}O_6$	421.1651	419.1495	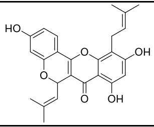
38	Cyclomulberrochromene	$C_{25}H_{22}O_6$	419.1495	417.1338	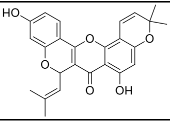
39	Cudranin	$C_{29}H_{26}O_{10}$	535.1599	533.1456	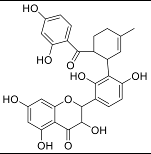
40	Morusin	$C_{25}H_{24}O_6$	421.1651	419.1495	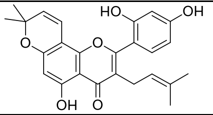
41	Moracin D	$C_{19}H_{16}O_4$	309.1127	307.0970	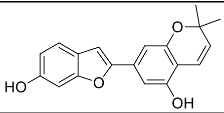
42	Moracin M	$C_{14}H_{10}O_4$	243.0567	241.0501	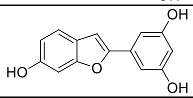
43	Betulinic acid	$C_{30}H_{48}O_3$	457.3682	455.3525	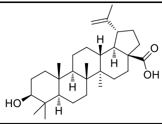

44	Kuwanon G	$C_{40}H_{36}O_{11}$	693.2336	691.2179	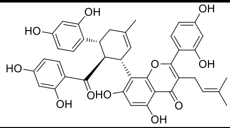
45	Mulberrofuran G	$C_{34}H_{26}O_{8}$	563.1706	561.1549	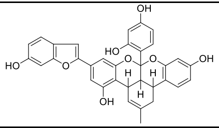
47	Soroceal B	$C_{27}H_{22}O_{7}$	459.1444	457.1287	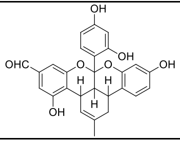
48	Sanggenol Q	$C_{25}H_{28}O_{6}$	425.1964	423.1808	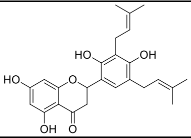
49	Mulberrofuran C	$C_{34}H_{28}O_{9}$	581.1812	579.1655	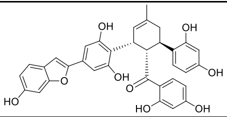
50	Sanggenol P	$C_{30}H_{36}O_{6}$	493.2590	491.2434	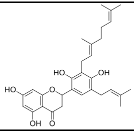
51	Licoflavone C	$C_{20}H_{18}O_{5}$	339.1232	337.1076	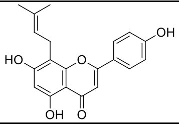
52	Morusinol	$C_{25}H_{26}O_{7}$	439.1757	437.1600	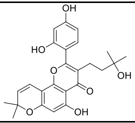
53	Sanggenon N	$C_{25}H_{26}O_{6}$	439.1751	437.1600	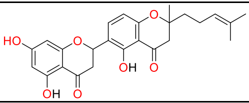
54	(2S)-4′-hydroxy-7-meth-oxy-8-prenylflavan	$C_{21}H_{24}O_{3}$	325.1804	323.1647	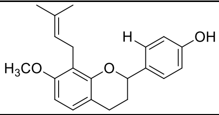
55	2′,7-dihydroxy-4′-methoxy-8- prenylflavan	$C_{21}H_{24}O_{4}$	341.1753	339.1596	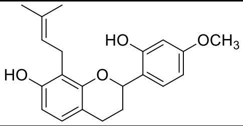
56	Brosimine B	$C_{20}H_{22}O_{3}$	311.1647	309.1491	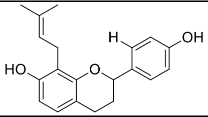
58	Morachalcone B	$C_{20}H_{20}O_{5}$	339.1232	337.1076	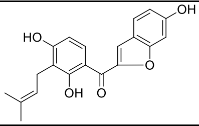
59	moracin C	C19H18O4	311.1283	309.1127	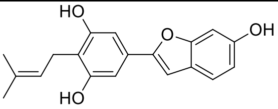
60	7-hydroxycoumarin	$C_{9}H_{6}O_{3}$	163.0395	161.0239	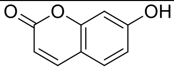

61	Scopoletin	$C_{10}H_8O_4$	193.0501	191.0344	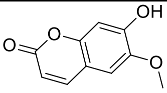
62	Sigmoidin A	$C_{25}H_{28}O_6$	425.1964	423.1808	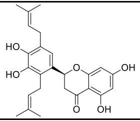
63	Ursolic acid	$C_{30}H_{48}O_3$	457.3682	455.3525	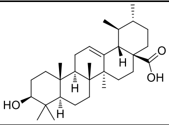
64	Fagomine	$C_6H_{13}NO_3$	148.0974		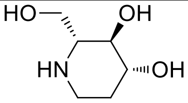
65	1,4-dideoxy-1,4-iminno-d-arabinitol	$C_5H_{11}NO_3$	134.0817		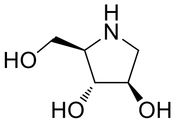
66	Zarzissine	$C_5H_5N_5$	136.0623		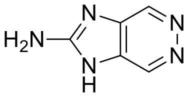
67	Amidinoproline	$C_6H_{11}N_3O_2$	158.0930		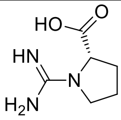
68	2-methyl-hydrazinecarbox-yli cacid	$C_2H_6N_2O_2$	91.0508		$H_3C-NH-NH-COOH$
69	Norscopolamine	$C_7H_{12}NO_4$	176.0923		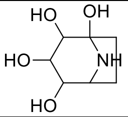
70	Grateloupinami	$C_5H_{11}N_3O_2$	146.0930		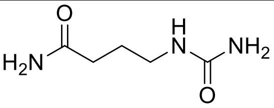
71	SanggenolP	$C_{30}H_{36}O_6$	493.2590	491.2434	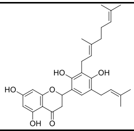
72	Mulberrofuran G	$C_{34}H_{26}O_8$	563.1706	561.1549	
73	Sanggenol A	$C_{25}H_{28}O_6$	425.1964	423.1808	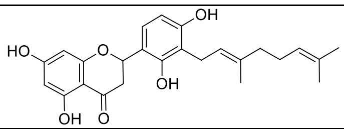
74	Sanggenol L	$C_{25}H_{26}O_6$	423.1808	421.1651	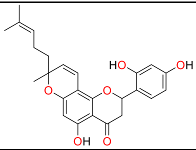
75	Cyclocommunol	$C_{20}H_{16}O_6$	353.1025	351.0869	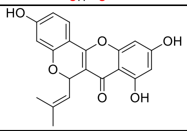
76	Morusone	$C_{25}H_{22}O_7$	435.1444	433.1287	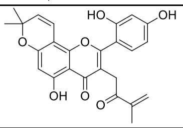

77	Steppogenin	$C_{15}H_{12}O_6$	289.0712	287.0556	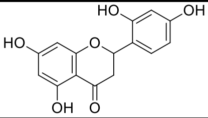
78	Dihydrokaempferol	$C_{15}H_{12}O_6$	289.0712	287.0556	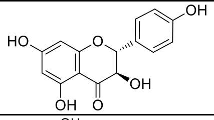
79	Eriodictyol	$C_{15}H_{12}O_6$	289.0712	287.0556	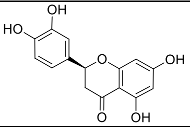
80	2,4-dihydroxybenzoic acid	$C_7H_6O_4$	155.0344	153.0188	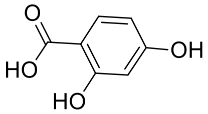
81	*p*-coumaric acid	$C_9H_8O_3$	165.0552	163.0395	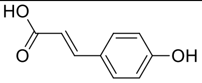
82	Moracin D	$C_{20}H_{18}O_4$	323.1283	321.1127	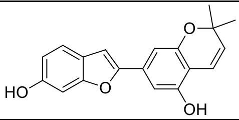
82	Moracin J	C15H12O5	273.0763	271.0606	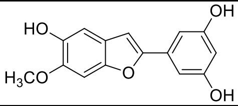
82	Moracin B	$C_{20}H_{18}O_4$	287.0919	285.0763	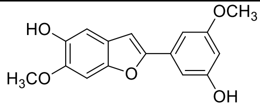
83	2,4,2′,4′-tetrahydroxy-chalcone	$C_{15}H_{12}O_5$	273.0763	271.0606	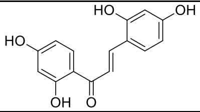
84	5,7,2′,4′-tetrahydroxy-3-methoxyflavone	$C_{17}H_{14}O_6$	315.0869	313.0712	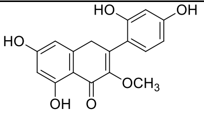
85	(2E)-1-[2,3-dihydro-4-hydroxy-2-(1-methylethenyl)-5-benzofuranyl]-3-(4-hydroxyphenyl)-1-propanone	$C_{20}H_{18}O_4$	323.1283	321.1127	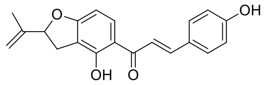
86	Quercetin	$C_{15}H_{10}O_7$	303.0505	301.0348	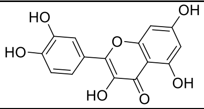
87	3-O-(6′′-O-acetyl)-β-D-glucopyranoside	$C_{23}H_{22}O_{13}$	507.1139	506.1060	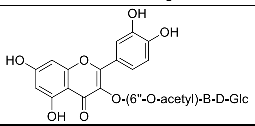
88	Isouercetin	$C_{21}H_{20}O_{12}$	465.1033	463.0877	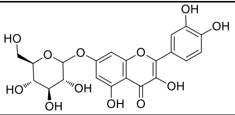
89	Quercetin 3,7-di-O-b-D-glucopyranoside	$C_{27}H_{30}O_{17}$	627.1561	625.1405	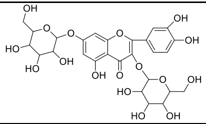

90	Kaempferol 3-O-b-D-glucopyranoside	$C_{21}H_{20}O_{11}$	449.1084	447.0927	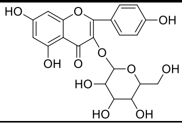
91	5,7,3′-trihydroxy-flavanone-4′-O-b-D-glucopyranoside	$C_{21}H_{20}O_{11}$	449.1084	447.0927	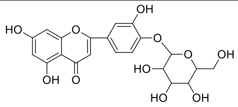
92	5,7,4′-trihydroxy-flavanone-3′-O-b-D-glucopyranoside	$C_{21}H_{20}O_{11}$	449.1084	447.0927	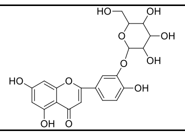
93	Jaboticabin	$C_{16}H_{14}O_8$	335.0767	333.0610	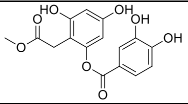
94	2-O-(3,4-dihydroxybenzoyl)-2,4,6-trihydroxyphenylacetic acid	$C_{15}H_{12}O_8$	321.0610	319.0454	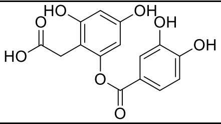
95	p-hydroxybenzoic acid	$C_7H_6O_3$	139.0395	137.0239	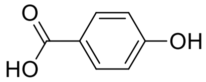
96	Protocatechuic acid	$C_7H_6O_4$	155.0344	153.0188	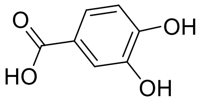
97	vanillic acid	$C_8H_8O_4$	169.0501	167.0344	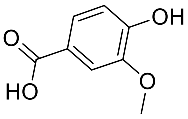
98	vanillic acid methyl ester	$C_8H_8O_4$	169.0501	167.0344	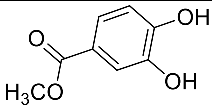
99	protocatechuic acid ethyl ester	$C_9H_{10}O_4$	183.0657	181.0501	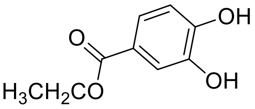
100	4-hydroxyphenylacetic acid methyl ester	$C_9H_{10}O_3$	167.0708	165.0552	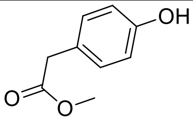
101	5,7-dihydroxychromone	$C_9H_6O_4$	179.0344	177.0188	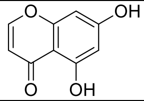
102	Tyrosol	$C_8H_{10}O_2$	139.0759	137.0603	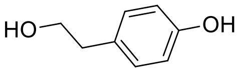
103	Pyrocatechol	$C_6H_6O_2$	111.0446	109.0290	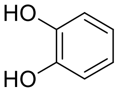
104	Acetic acid	$C_2H_4O_2$	61.0290	59.1033	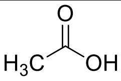
105	Alanine	$C_3H_7NO_2$			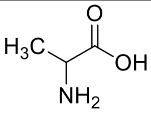
106	Asparagine	$C_4H_7NO_4$			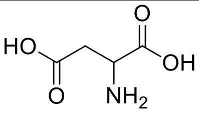

107	Choline	$C_5H_{13}NO_4^+$			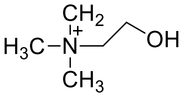
108	Fumaric acid	$C_4H_4O_4$			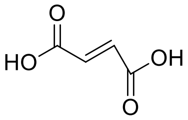
109	GABA	$C_4H_9NO_4$			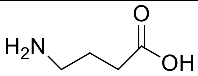
110	Glucose	$C_6H_{12}O_5$	165.0763	163.0606	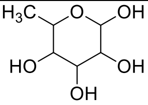
111	Proline	$C_5H_9NO_2$	116.0712	114.0555	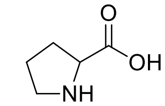
112	Succinic acid	$C_4H_6O_4$	119.0344	117.0188	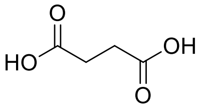
113	Sucrose	$C_{12}H_{22}O_{11}$	343.1240	341.1084	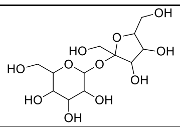
114	Trigonelline	$C_7H_7NO_2$	138.0555	136.0399	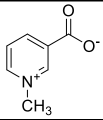
115	Kaempferol 3-*O*-rutinoside	$C_{27}H_{30}O_{15}$	595.1663	593.1506	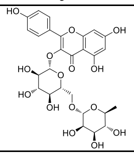
116	5-*O*-caffeoylquinic acid	$C_{16}H_{18}O_{19}$	355.1029	353.0873	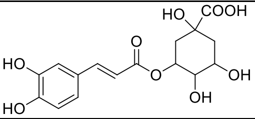
117	3-*O*-(6′′-*O*-a-rhamnopyranosyl-b-glucopyranoside)	$C_{27}H_{31}O_{15}$	595.1663		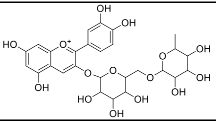
118	Cyanidin 3-*O*-b-glucopyranoside	$C_{21}H_{21}O_{11}$	449.1078		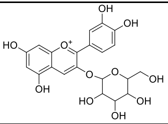
119	Cyanidin 7-O-b-glucopyranoside	$C_{21}H_{21}O_{11}$	449.1078		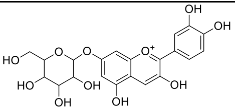
120	Artoindonesianin O	$C_{20}H_{20}O_4$	325.1440	323.1283	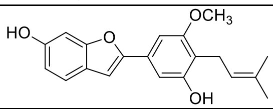
121	Loliolide	$C_{11}H_{16}O_3$	197.1178	195.1021	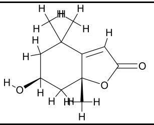
123	Steppogenin-7,4′-di- O-β-D-glucosiade	$C_{27}H_{32}O_{16}$	613.1769	611.1612	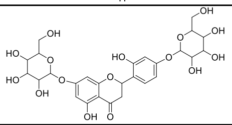

124	Aldehyde	$C_6H_{12}O_3$	133.0865	131.0708	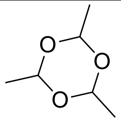
125	DL-Proline	$C_5H_9NO_2$	116.0712		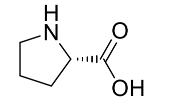
126	Phenprobamate	$C_{10}H_{13}NO_2$	180.1025		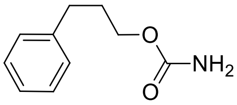
127	Luteolin	$C_{15}H_{10}O_6$	287.0556	285.0399	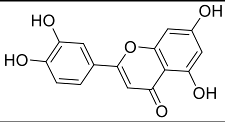
128	Butein	$C_{15}H_{12}O_5$	273.0763	271.0606	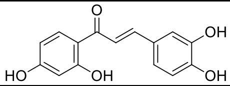
129	Hyperoside	$C_{21}H_{20}O_{12}$	465.1033	463.0877	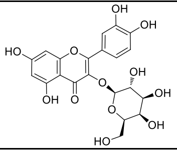
130	Rubraflavone a	$C_{25}H_{26}O_5$	407.1858	405.1702	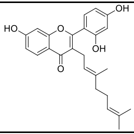
131	Naringenin	$C_{15}H_{12}O_5$	273.0763	271.0606	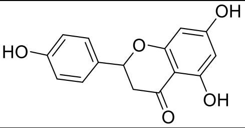
132	Liriodendrin	$C_{34}H_{46}O_{18}$	743.2762	741.2606	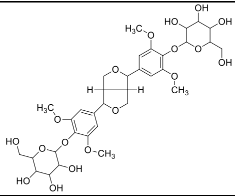
133	Vanillin	$C_8H_8O_3$	153.0552	151.0395	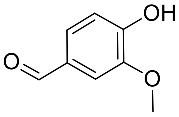
134	Steppogenin-7,4'-di-O-β-D-glucosiade	$C_{27}H_{32}O_{16}$	613.1769	611.1612	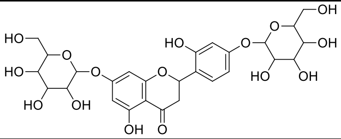
135	5-(hydroxymethyl)-1H-pyrrole-2-carboxaldehyde	$C_6H_7NO_2$	126.0555	124.0399	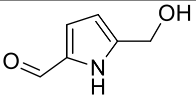
136	2-formyl-1H-pyrrole1-butanoic acid	$C_9H_{11}NO_3$	182.0817		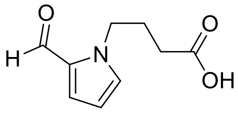
137	2-formyl-5-(hydroxymethyl)-1H-pyrrole-1- butanoic acid	$C_{10}H_{13}NO_4$	212.0923		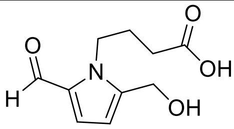

138	2-formyl-5-(methoxymethyl)-1H-pyrrole1-butanoic acid	$C_{11}H_{15}NO_4$	226.1079		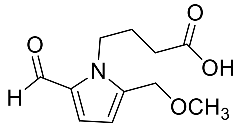
139	Morrole A	C14H21NO5	+Na 306.1317 +H 284.1498		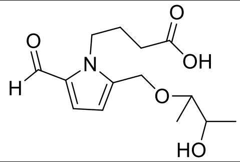
140	Albasins A	$C_{44}H_{44}O_9$	717.3064	715.2907	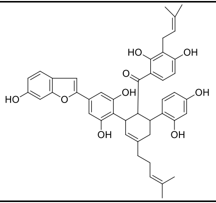
141	Albasin B	$C_{34}H_{28}O_8$	565.1862	563.1706	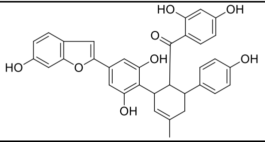
142	Albasin C	$C_{24}H_{26}O_5$	395.1858	393.1702	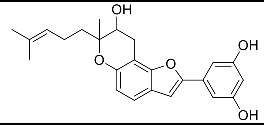
143	Albasin D	$C_{25}H_{24}O_5$	405.1702	403.1545	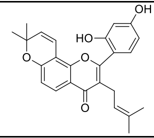
144	Kuwanon E	$C_{25}H_{28}O_6$	425.1964	423.1808	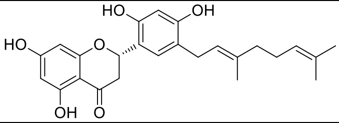
145	Sanggenon U	$C_{30}H_{38}O_7$	511.2696	509.2539	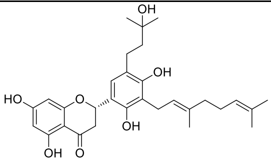
146	Sanggenon W	$C_{25}H_{26}O_6$	422.1729	420.1573	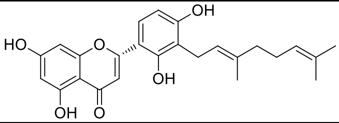
147	Kuwanon S	$C_{25}H_{26}O_5$	407.1858	405.1702	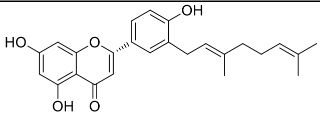
148	Euchrenone a7	$C_{20}H_{20}O_5$	341.1389	339.1232	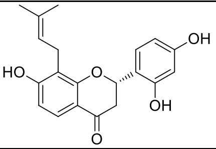
149	Sanggenon V	$C_{25}H_{24}O_6$	421.1651	419.1495	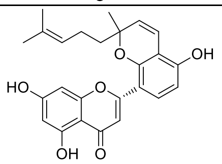

150	(7″R)-(−)-6-(7″-Hydroxy-3″,8″-dimethyl-2″,8″-octadien-1″-yl)apigenin	$C_{25}H_{26}O_6$	423.1808	421.1651	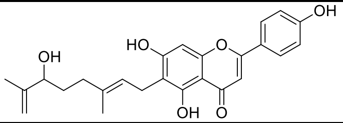
151	10-Oxomornigrol F	$C_{25}H_{24}O_7$	437.1600	435.1444	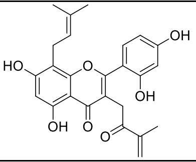
152	Ramumorin A	$C_{40}H_{38}O_{10}$	679.2543	677.2387	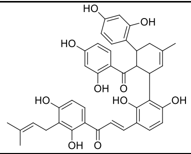
153	Ramumorin B	$C_{39}H_{36}O_9$	649.2438	647.2281	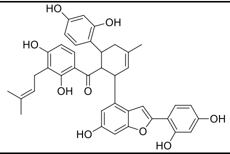
154	kuwanon G	$C_{40}H_{38}O_{11}$	695.2492	693.2336	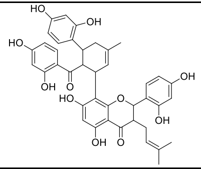
155	(4S,7S,8R)-Trihydroxyoctadeca-5Z-enoic acid	$C_{18}H_{34}O_5$	449331.2484	329.2328	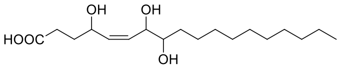
156	β-amyrin glucopyranoside	$C_{36}H_{60}O_6$	589.4468	587.4312	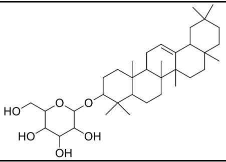
157	2′,4′,7-trihydroxyflavone	$C_{15}H_{12}O_5$	273.0763	271.0606	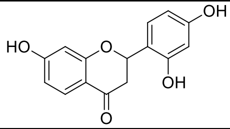
158	3,4′,5,7- tetrahydroflavone	$C_{15}H_{12}O_6$	289.0712	287.0556	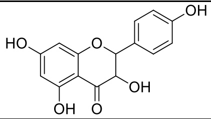
159	5-(5-hydroxybenzofuran-2-yl)benzen-1,3-diol	$C_{14}H_{10}O_4$	243.0567	241.0501	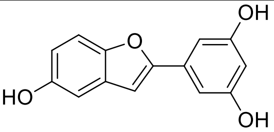
160	5-methoxymorican M	$C_{20}H_{18}O_4$	287.0919	285.0763	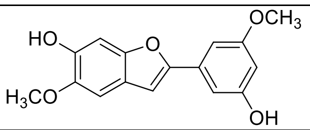
161	Norartocarpetin	$C_{15}H_{10}O_6$	287.0556	285.0399	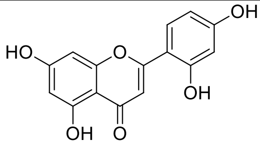
162	Albanin A	C20H18O6	355.1182	353.1025	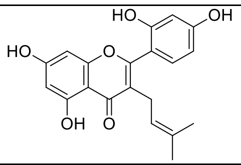

163	Isohamnetin	$C_{16}H_{12}O_7$	317.0661	315.0505	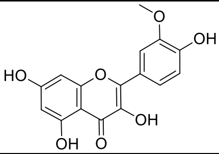
164	Mornigrol F	$C_{25}H_{26}O_7$	439.1757	437.1600	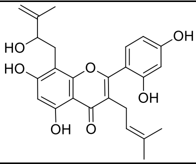
165	Cudraflavone B	$C_{25}H_{24}O_6$	421.1651	419.1495	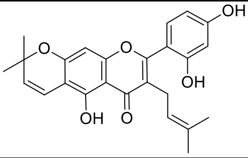
166	Isoliquiritigenin	$C_{15}H_{12}O_4$	257.0814	255.0657	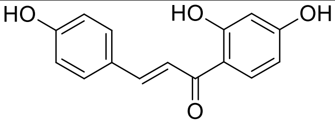
167	Mornigrol E	$C_{25}H_{26}O_7$	439.1757	437.1600	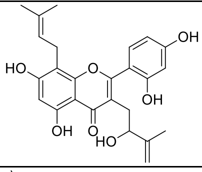
168	Moracin G	$C_{19}H_{16}O_4$	309.1127	307.0970	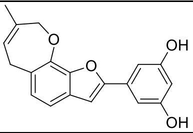
169	2,2′,4,4′-tetrahydroxychal-cone	$C_{15}H_{12}O_5$	273.0763	271.0606	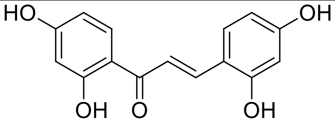
170	Mulberrofuran A	$C_{25}H_{28}O_4$	393.2066	391.1909	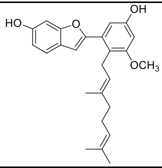
171	2-(2,4-dihydrophenyl)-5-hydroxy-8-(hydroxyme-thyl)-8-methyl-3-(3-me-thyl-2-butenyl)-(9CI)	$C_{25}H_{24}O_7$	437.1600	435.1444	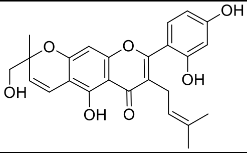
172	3- methoxyquercetin	$C_{16}H_{12}O_7$	317.0661	315.0505	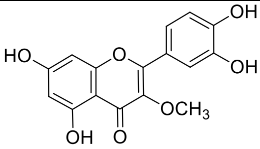
173	Norartocarpanone	$C_{15}H_{12}O_6$	289.0712	287.0556	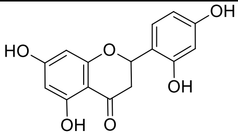
174	5-methoxymorican M	$C_{16}H_{14}O_5$	287.0919	285.0763	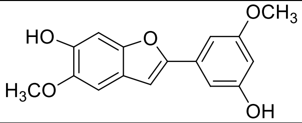

175	Mulberranol	$C_{25}H_{26}O_7$	439.1757	437.1600	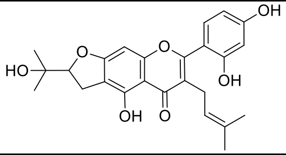
176	p-hydroxybenzadehyde	$C_7H_6O_2$	123.0446	121.0290	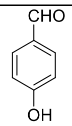
177	p-hydroxyphenol	$C_6H_6O_2$	111.0446	109.0290	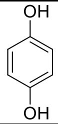
178	p-courmaric acid	$C_9H_8O_3$	165.0552	163.0395	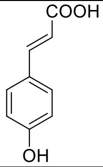
179	2′,3,4′,5,5′-pentahydroxy-cis-stilbene	$C_{14}H_{12}O_3$	229.0865	227.0708	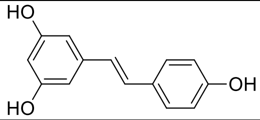
180	Cudraflavone A	$C_{25}H_{22}O_6$	419.1495	417.1338	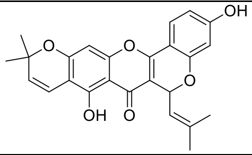
181	Albafuran C	$C_{34}H_{28}O_9$	581.1812	579.1655	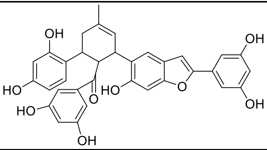
182	Mulberrofuran G	$C_{34}H_{26}O_8$	563.1706	561.1549	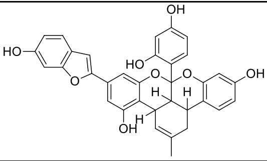
183	Uvaol	$C_{30}H_{50}O_2$	443.3889	441.3733	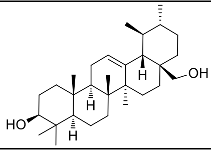
184	Guangsangon A	$C_{41}H_{42}O_9$	679.2907	677.2751	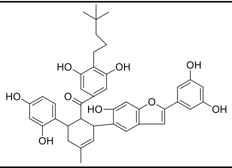
185	Kuwanon P	$C_{34}H_{30}O_9$	583.1968	581.1812	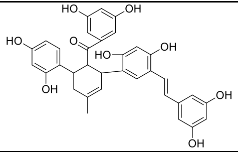
186	Guangsangon B	$C_{34}H_{30}O_8$	567.2019	565.1862	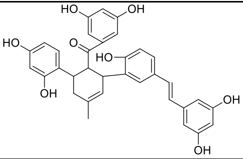

187	Kuwanon X	$C_{34}H_{30}O_9$	583.1968	581.1812	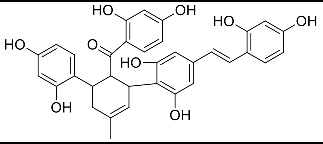
188	Guangsangon C	$C_{35}H_{30}O_{10}$	611.1917	609.1761	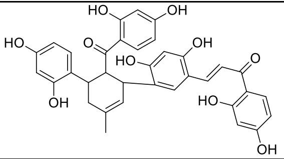
189	Guangsangon D	$C_{35}H_{30}O_{10}$	611.1917	609.1761	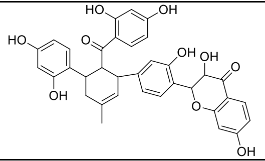
190	Guangsangon E	$C_{39}H_{36}O_9$	649.2438	647.2281	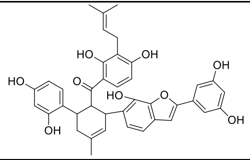
191	6,7-dimethylesculetin	$C_{11}H_{10}O_4$	207.0657	205.0501	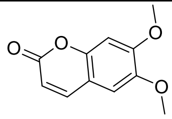
192	2′,4′-dihydroxy-7′- methoxy-8-prenylflavan	$C_{21}H_{24}O_4$	341.1753	339.1596	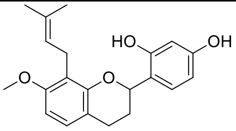
193	4-hydroxybenzaldehyde	$C_7H_6O_2$	123.0446	121.0290	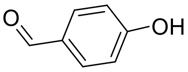
194	(+)-demethoxypinoresinol	$C_{19}H_{20}O_5$	329.1389	327.1232	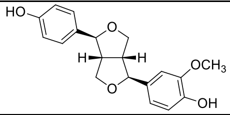
195	(+)-pinoresinol	$C_{20}H_{22}O_6$	359.1495	357.1338	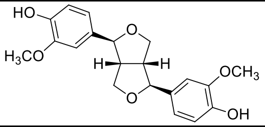
196	Umbellic acid	$C_9H_8O_4$	181.0501	179.0344	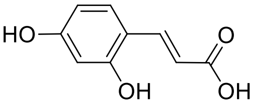
197	Linoleic acid	$C_{18}H_{32}O_2$	281.2481	279.2324	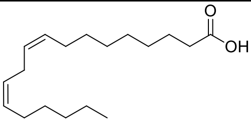
198	Methyl linoleate	$C_{19}H_{34}O_2$	295.2637	293.2481	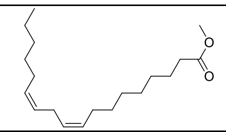
199	(9S, 10E, 12E)-9-hydroxy-10,12-octadecadienoic acid	$C_{19}H_{34}O_3$	311.2586	309.2430	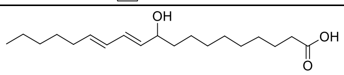
200	N-(N-benzoyl-L-phenylalanyl-L-phenylalanol	$C_{25}H_{26}N_2O_3$	403.2022	401.1865	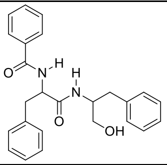

201	Isomoracin D	$C_{20}H_{18}O_4$	323.1283	321.1127	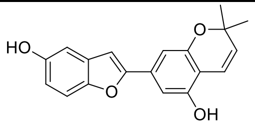
202	Sanggenol O	$C_{25}H_{24}O_6$	421.1651	419.1495	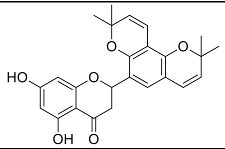
203	2,3-trans-dihydromorin	$C_{15}H_{12}O_7$	305.0661	303.0505	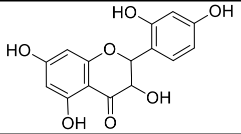
204	Sanggenon J	$C_{25}H_{26}O_6$	423.1808	421.1651	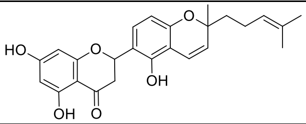
205	Sanggenon F	$C_{20}H_{18}O_6$	355.1182	353.1025	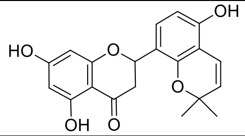
206	3'-Geranyl-3-prenyl-2',4',5,7-tetrahydroxyflavone	$C_{30}H_{34}O_6$	491.2434	489.2277	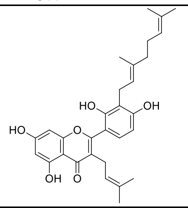
207	Kuwanon J	$C_{35}H_{30}O_{11}$	627.1861	625.1710	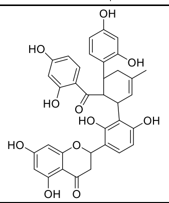
208	Kuwanon A	$C_{25}H_{24}O_6$	421.1651	419.1495	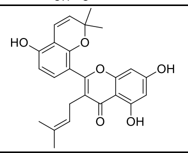
209	Kuwanon B	$C_{25}H_{24}O_6$	421.1651	419.1495	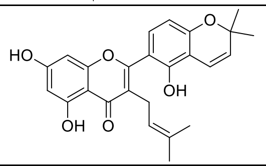
210	Kuwanol A	$C_{34}H_{28}O_8$	565.1862	563.1706	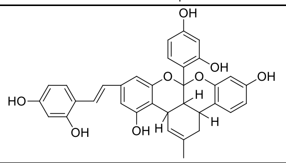

Table S2 The blood glucose level (mmol/L) of experimental mice.

Groups	0 Days	7 Days	14 Days	21 Days	28 Days
NC	5.37 ± 0.57	5.34 ± 0.93	5.56 ± 0.45	5.23 ± 0.50	5.28 ± 0.74
MC	32.38 ± 1.87**	32.19 ± 1.68**	32.56 ± 1.94**	33.12 ± 1.88**	33.23 ± 1.28**
Acarbose (AB)	33.08 ± 0.56	24.12 ± 0.79#	24.56 ± 0.43#	23.78 ± 0.78#	22.21 ± 0.73#
Morusin (MS)	31.45 ± 1.23	28.12 ± 0.95	27.45 ± 2.13	25.78 ± 1.95	23.45 ± 2.13#
RM	32.08 ± 1.27	27.26 ± 0.47	25.12 ± 0.97#	22.21 ± 2.03#	20.88 ± 1.82##
LM	31.17 ± 0.78	28.46 ± 0.12	28.67 ± 0.45	28.12 ± 0.67	27.33 ± 1.34
TM	33.32 ± 1.98	26.34 ± 1.21	26.45 ± 1.01	26.34 ± 1.45	24.74 ± 1.02#
FM	32.08 ± 1.07	29.12 ± 1.11	29.45 ± 1.78	30.21 ± 1.56	30.45 ± 1.89

** $p < 0.01$ compared with the normal control group on the same days. # $p < 0.05$, ## $p < 0.01$ compared with the data at the 0-days; NC: normal group; MC: model group; AB: acarbose group (positive control, 50 mg/kg); MS: morusin group (50 mg/kg); RM: roots of *M. alba* group (200 mg/kg); LM: leaves of *M. alba* group (200 mg/kg); TM: twigs of *M. alba* group (200 mg/kg); FM: fruits of *M. alba* group (200 mg/kg).

Table S3 The body weight (g) of experimental mice

Group	Weight（*g*）				
	0 Days	7 Days	14 Days	21 Days	28 Days
NC	36.17 ± 1.21	38.25 ± 1.53	40.34 ± 1.23	45.32 ± 2.46#	55.23 ± 1.26##
MC	40.34± 4.21	43.25 ± 4.32	40.23 ± 3.22	36.11 ± 3.34**	32.22 ± 2.44**
Acarbose (AB)	40.29 ± 1.11	40.12 ± 1.34	41.23 ± 1.67	42.34 ± 1.65	42.64 ± 1.43
Morusin (MS)	38.25 ± 2.67	38.23 ± 3.23	38.34 ± 4.01	38.67 ± 3.45	39.44 ± 3.67
RM	35.23 ± 1.44	36.23 ± 1.45	37.34 ± 1.07	39.23 ± 1.47	45.12 ± 1.45#
LM	36.21± 3.45	37.34 ± 3.27	37.89 ± 3.07	38.23 ± 2.88	38.56 ± 3.53
TM	35.34 ± 3.09	36.32 ± 3.27	37.23 ± 3.07	38.12 ± 2.88	39.44 ± 3.53
FM	36.70 ± 1.07	36.34 ± 1.27	36.67 ± 1.07	35.12 ± 1.88	34.01 ± 1.53

** $p < 0.01$ compared with the normal control group on the same days. # $p < 0.05$, ## $p < 0.01$ compared with the data at the 0-days; NC: normal group; MC: model group; AB: acarbose group (positive control, 50 mg/kg); MS: morusin group (50 mg/kg); RM: roots of *M. alba* group (200 mg/kg); LM: leaves of *M. alba* group (200 mg/kg); TM: twigs of *M. alba* group (200 mg/kg); FM: fruits of *M. alba* group (200 mg/kg).

Table S4 The postprandial glycemia (mmol/L) of the normal mice.

**Time (min)**	**blood glucose (mmol/L)**			
	**NC**	**Acarbose (AB)**	**RM**	**TM**
0	4.28 ± 0.89	4.21 ± 1.21	4.07 ± 0.23	4.45 ± 0.28
30	13.21 ± 1.11	10.34 ± 0.23*	9.01 ± 0.47*	10.25 ± 1.11*
60	10.89 ± 1.23	9.45 ± 0.89	8.00 ± 0.35	9.12 ± 0.78
90	8.21 ± 0.67	8.45 ± 0.76	7.67 ± 0.67	8.12 ± 0.35
120	6.34 ± 0.54	6.78 ± 0.93	6.21 ± 0.34	6.33 ± 0.65

* $p < 0.05$ compared with the normal control group (NC). NC: normal group; AB: acarbose group (positive control, 50 mg/kg); RM: roots of *M. alba* group (200 mg/kg); TM: twigs of *M. alba* group (200 mg/kg).

Table S5 Trial groups, number of mice and dose design of anti-diabetic experiment.

Group	Number of Mice	Dose （mg/kg）
Normal control (NC)	10	--
Model control (MC)	10	--
Positive control (Acarbose)	10	50
Morusin	10	50
RM	10	200
LM	10	200
TM	10	200
FM	10	200
